# Using Bioinformatics Approach to Explore the Pharmacological Mechanisms of Multiple Ingredients in *Shuang-Huang-Lian*

**DOI:** 10.1155/2015/291680

**Published:** 2015-09-30

**Authors:** Bai-xia Zhang, Jian Li, Hao Gu, Qiang Li, Qi Zhang, Tian-jiao Zhang, Yun Wang, Cheng-ke Cai

**Affiliations:** ^1^School of Chinese Materia Medica, Beijing University of Chinese Medicine, Beijing 100102, China; ^2^School of Basic Medical Sciences, Beijing University of Chinese Medicine, Beijing 100029, China; ^3^Institute of Basic Research of Clinical Medicine, China Academy of Chinese Medical Sciences, Beijing 100700, China

## Abstract

Due to the proved clinical efficacy, Shuang-Huang-Lian (SHL) has developed a variety of dosage forms. However, the in-depth research on targets and pharmacological mechanisms of SHL preparations was scarce. In the presented study, the bioinformatics approaches were adopted to integrate relevant data and biological information. As a result, a PPI network was built and the common topological parameters were characterized. The results suggested that the PPI network of SHL exhibited a scale-free property and modular architecture. The drug target network of SHL was structured with 21 functional modules. According to certain modules and pharmacological effects distribution, an antitumor effect and potential drug targets were predicted. A biological network which contained 26 subnetworks was constructed to elucidate the antipneumonia mechanism of SHL. We also extracted the subnetwork to explicitly display the pathway where one effective component acts on the pneumonia related targets. In conclusions, a bioinformatics approach was established for exploring the drug targets, pharmacological activity distribution, effective components of SHL, and its mechanism of antipneumonia. Above all, we identified the effective components and disclosed the mechanism of SHL from the view of system.

## 1. Introduction

Traditional Chinese Medicine (TCM), one of the main items of complementary and alternative medicine, is a healthcare focused medical system. As the main characteristics, formula is the most important part which has been utilized for treating diseases and promoting the health of humans for thousands of years in TCM practice [1]. As known, herbal formula is the most important part which has been utilized for treating diseases and promoting the health of humans for thousands of years in TCM practice. In the formula, each herb contains many compounds that offer multitarget, multicomponent synergy, and multidimensional pharmacological actions. Taking these concerns, there is a considerable challenge for researchers to disclose the mechanisms of formula using conventional pharmacological methods. Fortunately, with the development of pharmaceutical chemistry, more and more public databases of Traditional Chinese Medicine were built, such as Traditional Chinese Medicine Integrated Database (TCMID), Traditional Chinese Medicine Information Database (TCM-ID), TCMGeneDIT, and Chinese Traditional Medicine Herbs Database [2, 3]. Based on these qualitative databases, some valuable information could be addressed by system biological technology to identify some mechanisms of herbal formula.

Shuang-Huang-Lian (SHL), one of the famous modern formulae prepared from three medicinal herbs including* Flos Lonicerae*,* Radix Scutellariae*, and* Fructus Forsythiae*, mainly has antibacterial, antivirus, and anti-inflammation activities, which is put into clinic for treatment of the diseases including acute respiratory tract infection, bacterial infection, and pneumonia [4]. Currently, SHL has been developed in a variety of dosage forms due to its proved clinical efficacy, for example, SHL capsule, SHL soft capsule, SHL tablet, SHL oral liquid, SHL mixture, and SHL injection. However, the in-depth researches on holistic and synergetic pharmacological mechanisms of SHL preparations were scarce, and the studies on the targets of multicomponents and pharmacological activity of SHL are necessary for setup.

**Figure 1:**
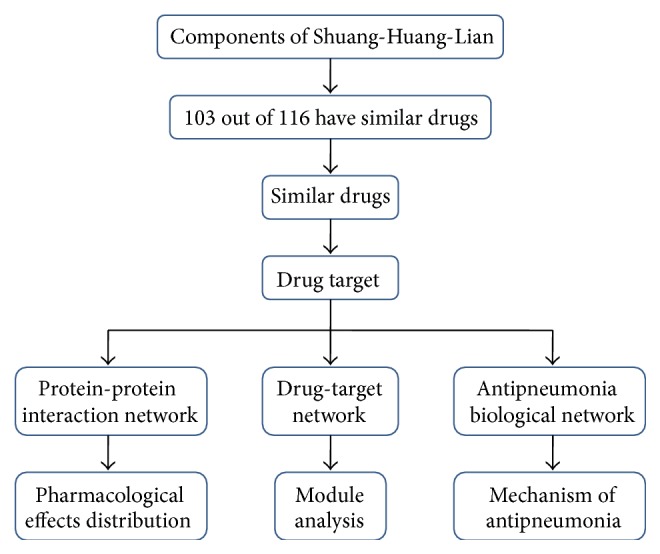
The workflow of SHL network construction and analysis.

In this study, a bioinformatics strategy, as shown in Figure 1, was adapted to explore the novel targets and activity of SHL. Hopefully, the approach could arouse a new paradigm for investigating and explaining the roles of herbal formulas.

## 2. Data Source and Methods

### 2.1. Data Sources

The components of SHL came from Traditional Chinese Medicine Basic Information Database of State Administration of Traditional Chinese Medicine of People's Republic of China (http://cowork.cintcm.com/engine/windex.jsp) and A Handbook on Analysis of the Active Composition in TCM [5] (Supplementary Table 1 in Supplementary Material available online at http://dx.doi.org/10.1155/2015/291680). The drug targeting proteins data comes from DrugBank database [6]. The context of network comes from REACTOME database [7]; protein-protein interactions (PPI) come from REACTOME database [7] and HPRD database [8]. Pharmacological effects data is collected from DrugBank database. The proteins related to pneumonia are derived from the SciClips database.

### 2.2. Similar Drugs of the SHL Components Are Predicted on DrugBank Database

Based on two-dimensional structural similarity, we could obtain the similar drugs (Supplementary Table 2) which exist in the DrugBank database by the module of the “ChemQuery Structure Search” in DrugBank (http://www.drugbank.ca/structures//search/small_molecule_drugs/structure). In order to obtain the most credible results, two conditions need to be satisfied: (1) the parameter of similarity threshold was higher than 0.6 and the other parameters' values were default; (2) the most similar drug to each component was retained.

### 2.3. Network Construction

In order to construct the TCM complex system model, identify effective cluster, and illustrate the mechanisms further, we put forward the directed TCM grammar systems (dTGS) [9, 10] which are based on the theory of Entity Grammar system [11]. dTGS is a tetrad, *G* = (*V*, *F*, *P*, *S*), whereas *V* is the character set representing basic element, *F* is a finite set of relations for *V*,  *V* and *F* were viewed as entity, *P* is a set of rules to deduce relationships between entities, and *S* is the starting entity. According to different objective, we can write different reasoning engine. Providing a starting condition (starting entity), dTGS can obtain the result of relationship among entities automatically. With these relationships among entities, we can construct networks. In this paper, we use dTGS as framework to find the relationships between components and other entities. The results, after reasoning and rearranging, were visualized with Cytoscape. Thus, we can construct the drug-target network and the antipneumonia biological network of SHL. The application of dTGS was more flexible compared to traditional network analysis methods. According to different objective, we can define different *V*, *F*, *P*, and *S*.

Due to the various objectives and data, the *V*, *F*, *P*, and *S* of the PPI network and antipneumonia biological network should be defined, respectively.

The *V*, *F*, *P*, and *S* of the PPI network are as follows:

(1) *V* = *V*_1_ ∪ *V*_2_ ∪ *V*_3_ ∪ *V*_4_:
 
*V*_1_ is the set of components of the corresponding herb, *V*_2_ is the similar drugs of components, *V*_3_ is the set of the targets of the drugs, and *V*_4_ is the set of rest proteins in the background network.
(2) *F* = {herbX(A), herbX(A, C), drugtarget(C, B), link(B, D), link(D, E)}:
 herbX(A) represents the components existing in the corresponding herb; herbX(A, C) represents the relationship of the components of the corresponding herb and its similar drug; drugtarget(C, B) represents the relationship of similar drug and its target; link(B, D) and link(D, E) represent the relationship of nodes in the background network which was prior knowledge.
(3) *P* = *P*_1_ ∪ *P*_2_ ∪ *P*_3_:
 
*P*_1_ = {herbX(A, C), drugtarget(C, B).⇒net(A, B, 1)}.
  
*P*_2_ = {net(A, B, 1), link(B, D).⇒net(A, D, 2)}.
 
*P*_3_ = {net(A, D, *N*), link(D, E), *N* < 10.⇒net(A, E, *N* + 1)}.
 
*P*_1_ are used to deduce the relations of chemical components and their targets. *P*_2_ and *P*_3_ are used to deduce the related proteins of components in PPI networks. “link(C, B)” as prior knowledge represents relations of nodes in PPI network. “net(A, B, *N*)” is the results which are obtained by reasoning engine; *N* is the cumulative distance from component A to protein E which is less than 10.
(4) *S* = *S*_1_ ∪ *S*_2_:
 
*S*_1_ is the set of entities with structure link(B, D) in background network for deduction. *S*_2_ is the starting point of reasoning with structure herbX(A).

The *V*, *F*, *P*, and *S* of the antipneumonia biological network is described by the following:

(1) *V* = *V*_1_ ∪ *V*_2_ ∪ *V*_3_ ∪ *V*_4_:
 
*V*_1_ is the set of targets of components of corresponding herb, *V*_2_ is the set of the pneumonia-related proteins, *V*_3_ is the set of rest proteins in the background network, and *V*_4_ is the set of rest proteins or small molecule metabolites in the background network.
(2) *F* = {link(A, B, *X*, *Y*), in(A), out(B), totalnet(A, B, *X*, *Y*), minnet(A, B, *X*, *Y*)}:
 In link(A, B, *X*, *Y*), A, B ∈ *V*_3_⁡, *X* ∈ {pos, neg}, *Y* ∈ *Z* ^*∗*^.
 In totalnet(A, B, *X*, *Y*), A ∈ *V*_1_∩*V*_3_, B ∈ *V*_3_, *X* ∈ {pos, neg}, *Y* ∈ *Z* ^*∗*^.
 In minnet(A, B, *X*, *Y*), A ∈ *V*_1_∩*V*_3_, B ∈ *V*_2_∩*V*_3_, *X* ∈ {pos, neg}, *Y* ∈ *Z* ^*∗*^.
 In(A) and out(B), A ∈ *V*_1_, B ∈ *V*_2_.
 link(A, B, *X*, *Y*) defines that A(protein) acts on B(protein) with an effect described in *X* and through *Y* reactions so that the distance number is *Y*. If process happens in one reaction, *Y* equals 1. In in(A) and out(B), A represents the targets of compounds, while B represents the pneumonia-related proteins. totalnet(A, B, *X*, *Y*) represents that A (target protein) affects B (pneumonia-related protein) with an effect described in *X* by reactions of *Y*. minet(A, B, *X*, *Y*) defines that A (target protein) can affect B (pneumonia-related protein) with an effect described in *X* by multiple pathway but we just retain the shortest path with *Y* reactions.
 (3) *P* = *P*_1_ ∪ *P*_2_ ∪ *P*_3_ ∪ *P*_4_ ∪ *P*_5_ ∪ *P*_6_ ∪ *P*_7_ ∪ *P*_8_ ∪ *P*_9_ ∪ *P*_10_ ∪ *P*_11_:
 
*P*_1_ = {link(A, B, *X*, *Y*), in(A)⇒totalnet(A, B, *X*, *M*)}.
 
*P*_2_ = {totalnet(A, B, pos, D), link(B, C, pos, E)⇒totalnet(A, C, pos, E + D)}.
 
*P*_3_ = {totalnet(A, B, pos, D), link(B, C, neg, E)⇒totalnet(A, C, neg, E + D)}.
 
*P*_4_ = {totalnet(A, B, neg, D), link(B, C, neg, E)⇒totalnet(A, C, pos, E + D)}.
 
*P*_5_ = {totalnet(A, B, neg, D), link(B, C, pos, E)⇒totalnet(A, C, neg, E + D)}.
 
*P*_6_ = {#min{D: totalnet(A, B, *X*, *Y*)} = *M*, totalnet(A, B, *X*, *Y*), out(B)⇒minnet(A, B, *X*, *M*)}.
 
*P*_7_ = {distance(C, B, *X*, *Y*)⇒length(*Y*)}.
 
*P*_8_ = {link(C, B, D), distance(_, B, _, _)⇒backward(C, B, D, 1)}.
 
*P*_9_ = {link(D, C, E), backward(C, B, D, *N*),  *M* = *N* + 1,  *N* < *Y*,  length(*Y*)⇒backward(D, C, E, F)}.
 
*P*_10_ = {distance(A, _, _, _), backward(A, C, *X*, *Y*)⇒forward(A, C, *X*, 1)}.
 
*P*_11_ = {forward(A, C, *X*, *N*), backward(C, D, E, F), *M* = *N* + 1, *N* = *Y* − F, length(*Y*)⇒forward(C, D, E, *M*)}.
 
*P*_1_ tags the starting point of derivation by in(A) from link(A, B, *X*, *Y*), and the tagged link(A, B, *X*, *Y*) is named as totalnet(A, B, *X*, *Y*). *P*_2_ ∪ *P*_3_ ∪ *P*_4_ ∪ *P*_5_ is the set of rules to deduce the eventual effects and distances of target proteins to other downstream proteins in the network. In link(A, B, *X*, *Y*), totalnet(A, B, pos, D), and minnet(A, B, *X*, *Y*), “*X*” and “pos” and “*Y*” and “D” represent the same data type, respectively, just because they located in the same position. *P*_2_ indicates that if the effect of A on B is positive, and the effect of B on C is positive too, then the effect of A on C is positive. Meanwhile, if A affects B through D reactions, and B affects C through E reactions, then the distance of A to C is D plus E. Similar derivations are defined in *P*_3_, *P*_4_, and *P*_5_. They may be used as many times as necessary to the final pneumonia-related protein. *P*_6_ is a rule used to identify the shortest distance from a target protein to a pneumonia-related protein when the paths between them are too complicated to analyze. In *P*_6_, if B is a pneumonia-related protein in totalnet(A, B, *X*, *Y*), then #min{D: totalnet(A, B, *X*, *Y*)} = *M* indicates that the shortest distance from A to B is *M*, which is extracted by minnet(A, B, *X*, *M*). In the construction of target network, *P*_6_ will be used as many times as necessary to the target protein pneumonia-related protein pairs. *P*_7_ ∪ *P*_8_ ∪ *P*_9_ ∪ *P*_10_ ∪ *P*_11_ were used to describe the detailed pathway from A to B with the clear steps of *Y*. The forward(C, D, E, *M*) was the final result used to construct the network. After connecting each of the forward(C, D, E, *M*), we could get the detailed pathway from A to B.
(4) *S* = *S*_1_ ∪ *S*_2_:
 
*S*_1_ is the set of entities with structure link(A, B, *X*, *Y*) in background network for deduction. *S*_2_ is the set of starting and end point proteins, described by in(A) and out(B).

## 3. Results and Discussion

### 3.1. Topology Analysis of SHL PPI Network

**Figure 2:**
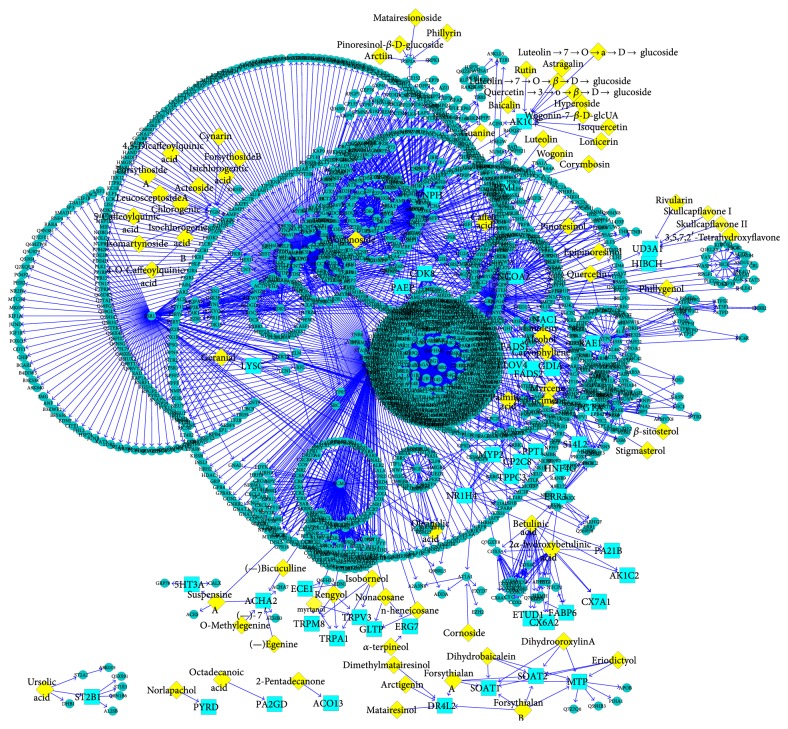
PPI Network of SHL. The components in SHL Network are yellow diamonds. The targets are shown as blue squares. In the PPI network, there are 92 components (Supplementary Table 3) acting on 129 targets which interact with 1787 proteins.

As shown in Figure 2 (clear node label can be seen in Figure 2), the SHL PPI network, there are 1953 nodes and 3112 edges. The network diameter is 12, which means the greatest distance between any pair of vertices is 12. The node degree distribution indicated that the PPI of SHL followed the power law with a degree exponent of 0.969 (*R*2 = 0.647). The Clustering coefficient is 0.322. The connected component is 1.0. Network centralization is 0.3112. And network heterogeneity as 6.017. These common topological parameters suggested that the network exhibited scale-free property and modular architecture.

### 3.2. Drug-Target Network

**Figure 3:**
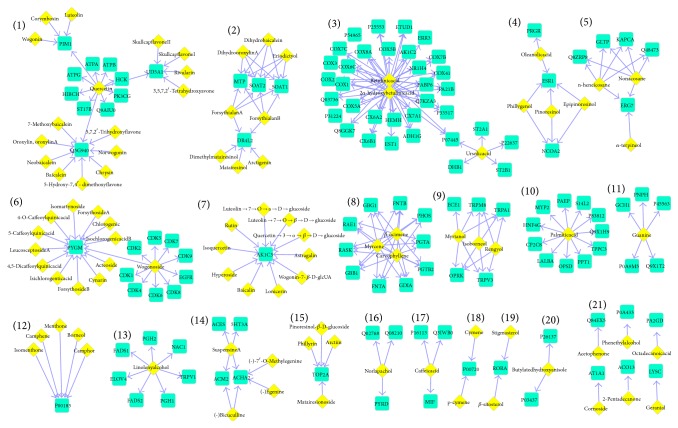
Drug-target Network of SHL. The components are yellow diamonds. The targets are shown as blue squares.

In order to express the relationship between SHL components more clearly, we constructed a drug-target network of SHL. As shown in Figure 3, we totally labeled the 21 modules. Most of them match the common targets. However, it may be worth nothing that some novel targets are detected, such as thirteen components of the module (6) act on the same targets, PYGM, which play an important role in regulation of cell cycle and cellular macromolecule metabolic process. These biological processes are closely related to cell proliferation and tumorigenesis (anticancer effect). Following the same strategy, it is easy to help us to understand the functional classifications of each SHL component based on the modules in drug-target network.

### 3.3. Pharmacological Effects Distribution of SHL

**Figure 4:**
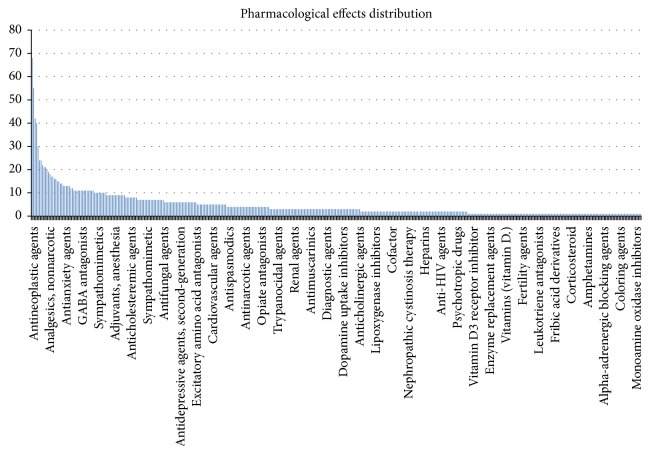
The pharmacological distribution of all 112 components in SHL.

In the PPI Network of SHL, we counted pharmacological effects of all the proteins; frequency statistical results are presented in Figure 4. The results indicate that five in top ten pharmacological effects of SHL are linked with antineoplastic agents, protein kinase inhibitors, enzyme inhibitors, growth inhibitors, and phytogenic antineoplastic agents, which suggested that SHL might directed against tumor effect. Furthermore, two pharmacological effects focused on antioxidants and anti-inflammatory agents, which could help tumor suppression. For sure, the novel pharmacological effects of SHL need further empirical data form bench to bedside.

### 3.4. The Anti-Pneumonia Mechanism of SHL

**Figure 5:**
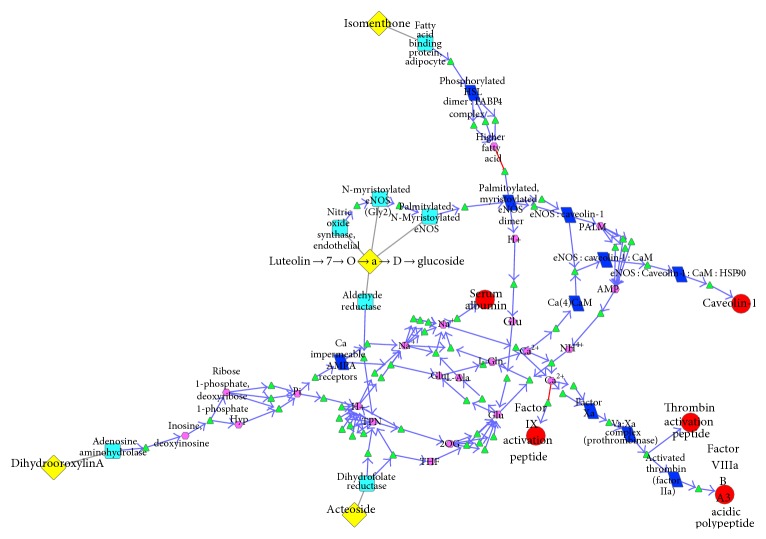
The antipneumonia biological network of SHL.

Using the data derived from the Reactome database as context, we constructed the biological network of the effective components of SHL acting on the pneumonia related targets. Circular node represents disease related targets, diamond node represents effective components of SHL, square node represents proteins involved in the pathway, parallelogram node represents complex, hexagon node represents micromolecule, and triangle node represents biological reaction. The edge with triangle arrow represents positive regulation, edge with T-shaped arrow represents negative regulation, and the undirected edge means the direction is uncertain (Figure 5). This biological network which included 4 components, 5 pneumonia related targets, and 26 subnetworks could overall exhibit the effective components and the mechanism of antipneumonia on a molecular level.

**Figure 6:**
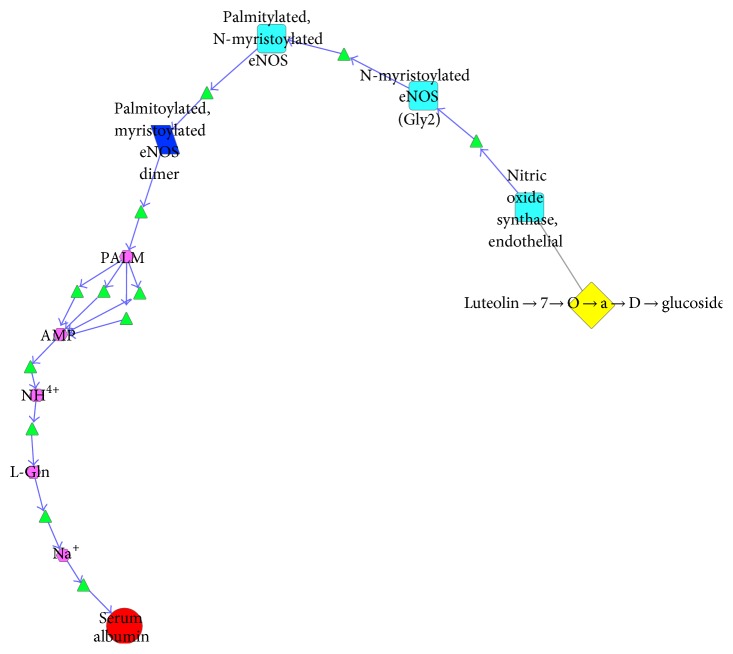
The antipneumonia biological pathway of luteolin-7-o-*α*-D-glucoside.

**Figure 7:**
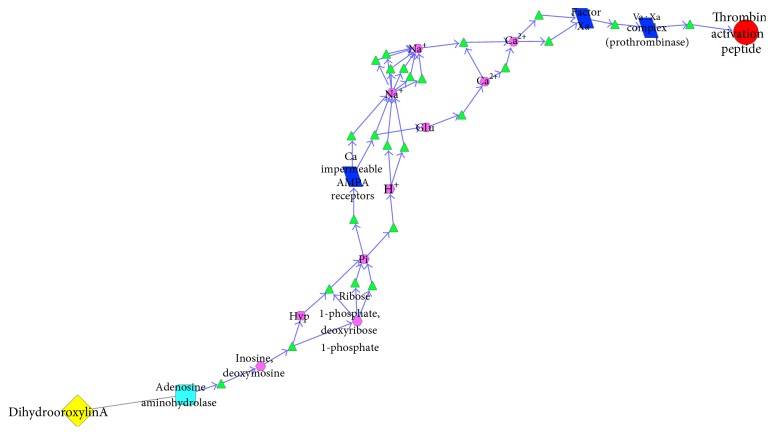
The antipneumonia biological pathway of dihydrooroxylin A.

This biological network included 26 subnetworks that 4 components that acted on 5 pneumonia related targets. All of these subnetworks intertwined to play the role of antipneumonia collectively and demonstrated the features of biological systems simultaneously, such as robustness, redundancy, crosstalk, and so forth. In order to clearly show the antipneumonia mechanism of SHL effective components, we could extracted 26 subnetworks, respectively. This paper took 2 extracted subnetworks as examples: the biological pathway where luteolin-7-o-*α*-D-glucoside acted on serum albumin (Figure 6) and the biological pathway where dihydrooroxylin A acted on thrombin activation peptide (Figure 7).

As target of luteolin-7-o-*α*-D-glucoside, endothelial nitric oxide synthase (eNOS) translocated from Golgi to caveolae after N-myristoylation and palmitoylation. With depalmitoylation of eNOS dimer, it produced PALM. After the reaction of PALM and CoASH, palmitic acid converted to palmitoyl-CoA, accompanyied with energy transformation. After a series of signal transduction of micromolecule (NH^4+^, L-Gln, Na^+^, etc.), the content of serum albumin changed. As the biomarker in the early stage, this pathway could elucidate the course of the change. That is, luteolin-7-o-*α*-D-glucoside acting on eNOS leads to the increase of vasopermeability and serum albumin influxed into the interval of capillaries, accelerated the speed of catabolism, and affected the content of serum albumin at last [12, 13].

Figure 7 shows the initial two steps of blood coagulation: the formation process of thrombin activation peptide and the activation of thrombin. As the target of dihydrooroxylin A, adenosine aminohydrolase was hydrolyzed, dephosphorylated, and oxidated. After some amino acid was taken up and GluR2 transferred, the Ca^2+^ impermeable AMPA receptor and aspartic acid receptor were activated and then extruded of Ca^2+^. Factor Xa was activated by Ca^2+^, and Ca^2+^ also could accelerate the combination of factors Xa and Va [14]. Factor Va had no activity itself, but it could enhance activity of factor Xa and accelerated the formation of thrombin [15]. Thrombin could accelerate the blood coagulation and wound healing, thus treating alimentary tract hemorrhage of severe pneumonia. In conclusion, dihydrooroxylin A acting on thrombin plays the role of antipneumonia by accelerating the hemostasis of the alimentary tract.

By summarizing the 26 subnetworks of SHL effective components cluster acting on pneumonia-related targets, we got 3 main pathways where SHL played the role of antipneumonia: (1) regulating the activity of caveolin-1 existing in the signal pathway of inflammatory and endothelial cells affects the response of inflammatory cell to the inflammation, further influencing the process of inflammation; (2) accelerating the blood coagulation and wound healing, thus treating alimentary tract hemorrhage of severe pneumonia; (3) affecting the content of serum albumin, promoting the repair of lung tissue, and enhancing the immune function.

Biological network shows 4 components could act on 5 targets within 10 biological reactions. Other components in SHL may also act on pneumonia-related targets, but the number of biological reactions will be more. When we defined the reactions as 15, biological network contained 5 components and 9 pneumonia-related targets. If the reaction was 20, 43 components and 13 targets were included in the biological network. The 4 components, by contrast, were more quickly and efficiently acting on pneumonia-related targets. Compared with the components in SHL which studied frequently, such as chlorogenic acid, forsythin, and baicalin, the content of four components within the network was lower, but they may play the same role of pneumonia treatment.

## 4. Conclusion

Based on the public databases of TCM, DrugBank database, and directed TCM grammar systems, we have built a PPI network, drug target network, and antipneumonia biological network of SHL. By the modules analysis, we predicted that SHL has the potential to be an antitumor candidate. This result may provide a novel clue for further experimental studies of SHL. In future, the predicted novel pharmacological effects of SHL should be further validated from bench to bedside. The antipneumonia biological network systematically explained the reason why SHL could play the role of antipneumonia and identified its effective components. All these are helpful to guide the quality control and further study of SHL.

Studies have already demonstrated that SHL had the pharmacological actions of antimicrobial, antiviral, antipyretic, anti-inflammatory, antioxidant, antiarrhythmic, and enhanced immunity [16–19], but they only focused on individual component, pharmacological action, or pathway. The component they studied could be extracted and was with a high content. Compared with these studies, our study could elucidate the mechanism holistically and on the molecular level. Meanwhile, enclosing the synergistic effect of various components and the cross of pathways, we not only addressed that luteolin-7-*α*-D-glucoside [20], dihydrooroxylin A [21, 22], and acteoside [23] played an anti-inflammatory role but also predicted that isomenthone could act on pneumonia-related targets through biological network of SHL, thus playing the role of antipneumonia.

The thought of this study accords with the network pharmacology, but the method we adopted is different from other related works [24, 25]. The whole process of other related works is carried out manually and the batch processing is impossible. With the characteristic of flexibility, dTGS could solve these problems effectively. Carrying out the TCM research under the guidance of network pharmacology is more quick and systematic than traditional methods. Meanwhile, dTGS provides a novel strategy for the study of TCM and other complex systems.

Because the research results were based on the inference of data included in the existing database or literature, and because we do not take into account the quantity of components and the specific interactional environment, this method still has some limitations. With further study of the complex systems, such as TCM formula and related disease, the data we used will be more complete and the results we got will be more precise and integrity.

## Supplementary Material

Supplementary Table 1 shows all components in SHL. In order to description the components in detail, the belonging herb, name, molecular formula, molecular weight, PubChem ID and smiles string of components are provided. Supplementary Table 2 shows the result of structural similarity between component and drug in DrugBank. It only shows the similarity coefficient which is higher than 0.6. Supplementary Table 3 shows the components which exist in PPI network. The belonging herb, name, molecular formula, molecular weight, PubChem ID and smiles string of components are also provided.
