# Supplementary material for: Using Bioinformatics Approach to Explore the Pharmacological Mechanisms of Multiple Ingredients in Shuang-Huang-Lian
Source: ScientificWorldJournal. 2015 Sep 30;2015:291680. doi: 10.1155/2015/291680 (PMC4606080; doi:10.1155/2015/291680)
Supplement: Supplementary file 1 — Supplementary Table 1 shows all components in SHL. In order to description the components in detail, the belonging herb, name, molecular formula, molecular weight, PubChem ID and smiles string of components are provided. Supplementary Table 2 shows the result of structural similarity between component and drug in DrugBank. It only shows the similarity coefficient which is higher than 0.6. Supplementary Table 3 shows the components which exist in PPI network. The belonging herb, name, molecular formula, molecular weight, PubChem ID and smiles string of components are also provided. [file 291680.f1.docx]

**S Table 1. All components in *Shuang-Huang-Lian***

| **Herb** | **Component** | **Molecular Formula** | **Molecular Weight** | **PubChem ID** | **Smiles string** |
| --- | --- | --- | --- | --- | --- |
| Scutellariae Radix | neobaicalein | C_17_H_14_O_6_ | 314.29 | 5320399 | O=C(C2)C(C(O)=CC(OC)=C1)=C1OC2C(C(O)=CC=C3)=C3O |
|  | chrysin | C_15_H_10_O_4_ | 254.24 | 5281607 | O=C(C(C(O)=CC(O)=C1)=C1O2)C=C2C3=CC=CC=C3 |
|  | skullcapflavoneⅠ | C_17_H_14_O_6_ | 314.29 | 5320399 | OC1=C2C(OC(C3=C(O)C=CC=C3)=CC2=O)=C(OC)C(OC)=C1 |
|  | baicalin | C_21_H_18_O_11_ | 446.35 | 64982 | OC([C@@H]1[C@@H](O)[C@H](O)[C@@H](O)[C@H](OC2=C(C(O)=C3C(OC(C4=CC=CC=C4)=CC3=O)=C2)O)O1)=O |
|  | baicalein | C_15_H_10_O_5_ | 270.24 | 5281605 | OC1=C(OC)C(O)=C(C(C=C(C3=CC=CC=C3)O2)=O)C2=C1 |
|  | wogonoside | C_22_H_20_O_11_ | 460.39 | 29927693 | OC([C@@H](O)[C@@H](O)[C@H](O)C(C(C)=O)(C1=C(OC)C(OC(C2=CC=CC=C2)=CC3=O)=C3C(O)=C1)O)=O |
|  | wogonin | C_16_H_12_O_5_ | 284.26 | 5281703 | COC1=C(OC)C(OC(C3=CC=CC=C3)=CC2=O)=C2C(OC)=C1 |
|  | 3,5,7,2'-tetrahydroxyflavone | C_15_H_10_O_6_ | 286.24 | 5281610 | OC1=CC=CC(O)=C1C3=C(O)C(C2=C(O)C=C(O)C=C2O3)=O |
|  | skullcapflavoneⅡ | C_19_H_18_O_8_ | 374.34 | 124211 | COC1=C(C(O)=C2C(C=C(OC2=C1OC)C3=C(C=CC=C3OC)O)=O)OC |
|  | oroxylin，oroxylinA | C_16_H_12_O_5_ | 284.26 | 5320315 | OC1=C(OC)C(O)=C(C(C=C(C3=CC=CC=C3)O2)=O)C2=C1 |
|  | dihydrooroxylinA | C_16_H_14_O_5_ | 286.3 | 177032 | OC1=C(OC)C(O)=C(C(CC(C3=CC=CC=C3)(C)O2)=O)C2=C1 |
|  | 5,7,2'-trihydroxyflavone | C_15_H_10_O_5_ | 270.24 | 5322064 | O=C1C=C(C3=CC=CC=C3O)OC2=C1C(O)=CC(O)=C2 |
|  | β-Sitosterol | C_35_H_60_O_2_ | 512.85 | 489644 | CC(C)[C@@](CC)([H])CC[C@@]([H])(C)[C@@]4([H])CC[C@@]3([H])[C@]2([H])CC=C1C[C@](O)([H])CC[C@@](C)1[C@]([H])2CC[C@@]34C |
|  | 7-methoxybaicalein | C_16_H_12_O_5_ | 284.27 | 6226 | O=C2C1=C(O)C(O)=C(OC)C=C1OC(C3=CC=CC=C3)=C2 |
|  | norwogonin | C_15_H_10_O_5_ | 270.24 | 5281674 | OC1=C2C(OC(C3=CC=CC=C3)=CC2=O)=C(C(O)=C1)O |
|  | dihydrobaicalein | C_15_H_12_O_5_ | 272.25 | 9816931 | OC1=C(C(O)=C2C(CC(OC2=C1)C3=CC=CC=C3)=O)O |
|  | eriodictyol | C_15_H_12_O_6_ | 288.25 | 440735 | O=C1C(C(O)=CC(O)=C2)=C2O[C@H](C3=CC(O)=C(O)C=C3)C1 |
|  | rivularin | C_18_H_16_O_7_ | 344.32 | 13889022 | COC1=CC=CC(O)=C1C(OC2=C3C(O)=CC(OC)=C2OC)=CC3=O |
|  | leucosceptosideA | C_36_H_48_O_19_ | 784.75 | 45027865 | OC1=C(OC)C=C(/C=C/C(O[C@H]2[C@H](OC3O[C@@H](C)[C@H](O)[C@@H](O)[C@H]3O)[C@@H](O)[C@H](OCCC4=CC=C(O)C(O)=C4)O[C@@H]2CO)=O)C=C1 |
|  | acteoside | C_29_H_36_O_15_ | 624.59 | 5281800 | OC1=CC=C(C=C1O)/C=C/C(O[C@@H]2[C@@H](CO)O[C@@H](OCCC3=CC(O)=C(C=C3)O)[C@H](O)C2OC4O[C@@H](C)[C@H](O)[C@@H](O)[C@H]4O)=O |
|  | isomartynoside | C_31_H_40_O_15_ | 652.64 | 6476337 | OC1=C(OC)C=CC(/C=C/C(OC[C@H]2O[C@@H](OCCC3=CC(OC)=C(O)C=C3)[C@H](O)[C@@H](O[C@@H]4O[C@@H](C)[C@H](O)[C@@H](O)[C@H]4O)[C@@H]2O)=O)=C1 |
|  | salidroside | C_14_H_20_O_7_ | 300.3 | 159278 | O[C@@H]1[C@@H](O)[C@H](O)[C@@H](CO)O[C@H]1OCCC2=CC=C(O)C=C2 |
|  | isoprene | C_5_H_8_ | 68.12 | 6557 | CC(C=C)=C |
|  | acetophenone | C_8_H_8_O | 120.15 | 7410 | CC(C1=CC=CC=C1)=O |
|  | menthone | C_10_H_18_O | 154.25 | 26447 | O=C1C[C@@H](CC[C@H]1C(C)C)C |
|  | isomenthone | C_10_H_18_O | 154.25 | 6986 | O=C1CC(C)CCC1C(C)C |
|  | beta-patchoulene | C_15_H_24_ | 204.35 | 101731 | CC(C(C1)CC2)(C)C2(C3=C1C(C)CC3)C |
|  | guanine | C_5_H_5_N_5_O | 151.13 | 764 | NC(N1)=NC(N=CN2)=C2C1=O |
|  | 2-tert-butyl-4-methoxyphenol | C_11_H_16_O_2_ | 180.24 | 8456 | OC1=CC=C(OC)C=C1C(C)(C)C |
|  | phenethyl alcohol | C_8_H_10_O | 122.16 | 6054 | OCCC1=CC=CC=C1 |
|  | isoborneol | C_10_H_18_O | 154.25 | 6321405 | CC1([C@@]2(C)CC[C@](C[C@@H]2O)1[H])C |
|  | caryophyllene | C_15_H_24_ | 204.35 | 5322111 | C/C(CCC1C(C)(C)CC12)=C\CCC2=C |
|  | octadecanoic acid | C_18_H_36_O_2_ | 284.48 | 5281 | CCCCCCCCCCCCCCCCCC(O)=O |
|  | quebrachol | C_29_H_50_O | 414.71 | 222284 | CC(C)[C@@](CC)([H])CC[C@@]([H])(C)[C@@]4([H])CC[C@@]3([H])[C@]2([H])CC=C1C[C@](O)([H])CC[C@@](C)1[C@]([H])2CC[C@@]34C |
|  | stigmasterol | C_29_H_48_O | 412.69 | 412.69 | C[C@@]2(C(C(C)/C=C/[C@H](CC)C(C)C)CC3)C3C1CC=C(C4)[C@@](CC[C@@H]4O)(C)C1CC2 |
|  | daucosterol | C_35_H_60_O_6_ | 576.85 | 5742590 | O[C@@H]1[C@@H](O)[C@H](O[C@@H]3CC2=CC[C@]([C@@]5([H])[C@]([C@]([C@H](C)CC[C@@H](CC)C(C)C)([H])CC5)(C)CC4)([H])[C@@]4([H])[C@](C)2CC3)O[C@H](CO)[C@H]1O.[O] |
| Forsythiae Fructus | limonene | C_10_H_16_ | 136.23 | 22311 | CC1=CC[C@H]([C@@](C)=C)CC1 |
|  | myrcene | C_10_H_16_ | 136.23 | 31253 | C/C(C)=C/CCC(C=C)=C |
|  | suspensine A | C_19_H_17_NO_4_ | 323.34 | 6770 | CN1CC2=C(C=CC3=C2OCO3)CC4=CC5=C(OCO5)C=C4C1 |
|  | α-pinene | C_10_H_16_ | 136.23 | 6654 | CC1(C)C2CC=C(C)C1C2 |
|  | camphene | C_10_H_16_ | 136.23 | 6616 | CC1(C)C2CCC(C2)C1=C |
|  | β-pinene | C_10_H_16_ | 136.23 | 14896 | CC1(C)C(CC2)([H])CC([H])1C2=C |
|  | p-cymene | C_10_H_14_ | 134.22 | 7463 | CC1=CC=C(C(C)C)C=C1 |
|  | (-)-7′-O-methylegenine | C_21_H_21_NO_6_ | 383.39 | 197775 | CN1[C@]([C@]2([H])C(C=CC3=C4OCO3)=C4C(OC)O2)([H])C5=CC6=C(OCO6)C=C5CC1 |
|  | γ-terpinene | C_10_H_16_ | 136.23 | 7461 | CC1=CCC(C(C)C)=CC1 |
|  | β-phellandrene | C_10_H_16_ | 136.23 | 11142 | CC(C)C(CC1)C=CC1=C |
|  | forsythoside D | C_19_H_28_O_11_ | 432.42 | 192437 | OC1=C(O)C=C(C(CO[C@H]2[C@H](O)[C@@H](O)[C@H](O)[C@@H](CO[C@H]3[C@H](O)[C@H](O)[C@@H](O)[C@H](C)O3)O2)([H])O)C=C1 |
|  | β-ocimene | C_10_H_16_ | 136.23 | 5281553 | C/C(C)=C/C/C=C(C)/C=C |
|  | 3-carene | C_10_H_16_ | 136.23 | 26049 | CC1=CCC2C(C(C)2C)C1 |
|  | cymene | C_10_H_14_ | 134.22 | 10812 | CC1=CC=C(C(C)C)C=C1 |
|  | carveol（p-Mentha-1,8-dien-6-ol） | C_10_H_16_O | 152.23 | 7438 | CC(C1CC=C(C)C(O)C1)=C |
|  | myrcene | C_10_H_16_ | 136.23 | 31253 | C/C(C)=C/CCC(C=C)=C |
|  | camphor | C_10_H_16_O | 152.23 | 2537 | CC1(C2=O)C(C)(C)C(C2)CC1 |
|  | geranial | C_10_H_16_O | 152.23 | 638011 | C/C(C)=C/CC/C(C)=C/C=O |
|  | 2-pentadecanone | C_15_H_30_O | 226.4 | 61303 | CCCCCCCCCCCCCC(C)=O |
|  | borneol | C_10_H_18_O | 154.25 | 64685 | C[C@@]12[C@@H](C)C[C@@H]([C@@](C)2C)CC1 |
|  | α-terpineol | C_10_H_18_O | 154.25 | 17100 | CC1=CCC(C(C)(O)C)CC1 |
|  | safrole | C_10_H_10_O_2_ | 162.19 | 5144 | C=CCC1=CC=C2C(OCO2)=C1 |
|  | linalool | C_10_H_18_O | 154.25 | 6549 | C/C(C)=C\CCC(C=C)(O)C |
|  | terpinen-4-ol | C_10_H_18_O | 154.25 | 11230 | CC1=CCC(O)(C(C)C)CC1 |
|  | norlapachol | C_14_H_12_O_3_ | 228.24 | 231114 | OC2=C(\C=C(C)/C)C(C1=C(C2=O)C=CC=C1)=O |
|  | pinocarveol | C_10_H_16_O | 152.23 | 102667 | OC1C(C(C2)C(C)(C)C2C1)=C |
|  | myrcenol | C_10_H_18_O | 154.25 | 10975 | C=CC(CCCC(C)(O)C)=C |
|  | myrtanol | C_10_H_18_O | 154.25 | 521314 | CC12CCC(C)(CO)C(C)(C2)C(C)1C |
|  | piperitol | C_10_H_18_O | 154.25 | 10282 | CC(C)C1CCC(C)=CC1O |
|  | methyl tetracosanoate | C_25_H_50_O_2_ | 382.66 | 75546 | CCCCCCCCCCCCCCCCCCCCCCCC(=O)OC |
|  | cornoside | C_14_H_20_O_8_ | 3084796 | 3084796 | C1=CC(C=CC1=O)(CCO[C@@H]2[C@H]([C@@H]([C@H]([C@@H](O2)CO)O)O)O)O |
|  | rengyol | C_8_H_16_O_3_ | 160.21 | 363707 | OC1(CCO)CCC(O)CC1 |
|  | salidroside | C_14_H_20_O_7_ | 300.3 | 159278 | O[C@@H]1[C@@H](CO)O[C@@H](OCCC2=CC=C(O)C=C2)[C@H](O)[C@@H]1O |
|  | forsythosideA(suspensaside) | C_29_H_36_O_15_ | 624.59 | 45358127 | OC1=CC=C(/C=C/C(O[C@H]2[C@H](O)[C@@H](O)[C@H](OCCC4=CC(O)=C(O)C=C4)O[C@@H]2CO[C@H]3[C@H](O)[C@H](O)[C@@H](O)[C@H](C)O3)=O)C=C1O |
|  | forsythosideB | C_34_H_44_O_19_ | 756.7 | 23928102 | OC1=C(O)C=C(CCO[C@H]2[C@H](O)[C@@H](O)[C@H](OC(/C=C/C4=CC(O)=C(O)C=C4)=O)[C@@H](CO[C@@H]3O[C@@H](C)[C@H](O)[C@@H](O)[C@H]3O)O2)C=C1 |
|  | β-Hydroxyacteoside | C_29_H_36_O_16_ | 640.59 | 10009317 | OC1=CC(/C=C/C(O[C@H]2[C@H](O[C@@H]3O[C@@H](C)[C@H](O)[C@@H](O)[C@H]3O)[C@@H](O)[C@H](OCC(O)C4=CC=C(O)C(O)=C4)O[C@@H]2CO)=O)=CC=C1O |
|  | acteoside | C_29_H_36_O_15_ | 624.59 | 5281800 | OC1=C(O)C=C(/C=C/C(O[C@H]2[C@H](OC3O[C@@H](C)[C@H](O)[C@@H](O)[C@H]3O)[C@@H](O)[C@H](OCCC4=CC(O)=C(O)C=C4)O[C@@H]2CO)=O)C=C1 |
|  | arctigenin | C_21_H_24_O_6_ | 372.41 | 64981 | COC1=CC=C(C[C@@H]2[C@@H](CC3=CC=C(O)C(OC)=C3)C(OC2)=O)C=C1OC |
|  | arctiin | C_27_H_34_O_11_ | 534.55 | 100528 | CC[C@@H]1[C@@H](O)[C@H](O)[C@@H](O)[C@H](OC2=C(OC)C=C(C[C@H]3C(OC[C@@H]3CC4=CC(OC)=C(OC)C=C4)=O)C=C2)O1 |
|  | matairesinol | C_20_H_22_O_6_ | 358.39 | 119205 | COC1=CC(C[C@@H]2[C@@H](CC3=CC=C(O)C(OC)=C3)COC2=O)=CC=C1O |
|  | matairesionoside | C_26_H_32_O_11_ | 520.53 | 486612 | COC1=C(O[C@@H]3O[C@H](CO)[C@@H](O)[C@H](O)[C@H]3O)C=CC([C@H]([H])C2[C@@]([H])(CC4=CC=C(O)C(OC)=C4)COC2=O)=C1 |
|  | dimethylmatairesinol | C_22_H_26_O_6_ | 386.44 | 1286 | COC1=C(OC)C=CC(CC2C(CC3=CC=C(OC)C(OC)=C3)COC2=O)=C1 |
|  | phillyrin | C_27_H_34_O_11_ | 534.55 | 44584288 | O[C@@H]1[C@@H](O)[C@H](OC2=CC=[C@@]([C@H]3OC[C@@]4([H])[C@@]([H])3CO[C@H]4[C@]5=CC(OC)=C(OC)C=C5)C=C2OC)O[C@H](CO)[C@H]1O |
|  | phillygenol | C_21_H_24_O_6_ | 372.41 | 3083590 | COC1=C(OC)C=[C@@]([C@@H]2OC[C@@]3([H])[C@@]([H])2CO[C@@H]3[C@]4=CC(OC)=C(O)C=C4)C=C1 |
|  | pinoresinol | C_20_H_22_O_6_ | 358.39 | 234817 | OC1=C(OC)C=[C@@]([C@@H]2OC[C@@]3([H])[C@@]([H])2CO[C@H]3[C@]4=CC(OC)=C(O)C=C4)C=C1 |
|  | pinoresinol-β-D-glucoside | C_26_H_32_O_11_ | 520.53 | 486614 | O[C@@H]1[C@@H](O)[C@H](OC2=CC=[C@@]([C@H]3OC[C@@]4([H])[C@@]([H])3CO[C@H]4[C@]5=CC(OC)=C(O)C=C5)C=C2OC)O[C@H](CO)[C@H]1O |
|  | epipinoresinol | C_20_H_22_O_6_ | 358.39 | 637584 | OC1=CC=[C@]([C@@H]2[C@@H](CO[C@H]3[C@]4=CC=C(O)C(OC)=C4)[C@@H]3CO2)C=C1OC |
|  | forsythialan A | C_20_H_22_O_7_ | 374.38 | 44453412 | OC1=CC=C(C([C@]2([H])[C@]([H])(CO)[C@@H]([C@]3=CC(O)=CC(OC)=C3)OC2)=O)C=C1OC |
|  | forsythialan B | C_20_H_22_O_7_ | 388.41 | 44453332 | COC1=CC(O)=C[C@]([C@H]2OC[C@]([H])(C(C3=CC=C(OC)C(OC)=C3)=O)[C@](CO)2[H])=C1 |
|  | betulinic acid | C_30_H_48_O_3_ | 456.7 | 64971 | O[C@H]1CC[C@@]2(C)[C@](CC[C@]3(C)[C@@]([H])2CC[C@]4([H])[C@](C)3CC[C@]5([C@](O)=O)[C@@]([H])4[C@H]([C@@](C)=C)CC5)([H])C(C)1C |
|  | oleanolic acid | C_30_H_48_O_3_ | 456.7 | 10494 | O[C@H]1CC[C@@]2(C)[C@](CC[C@]3(C)[C@@]([H])2CC=C4[C@](C)3CC[C@]5([C@](O)=O)[C@]([H])4CC(C)(C)CC5)([H])C(C)1C |
|  | β-amyrin acetate (beta-Amyrenol) | C_30_H_50_O | 426.72 | 73145 | O[C@H]1CC[C@@]2(C)[C@](CC[C@]3(C)[C@@]([H])2CC=C4[C@](C)3CC[C@]5(C)[C@]([H])4CC(C)(C)CC5)([H])C(C)1C |
|  | ursolic acid | C_30_H_48_O_3_ | 456.7 | 64945 | O[C@H]1CC[C@@]2(C)[C@](CC[C@]3(C)[C@@]([H])2CC=C4[C@](C)3CC[C@]5([C@@](O)=O)[C@]([H])4[C@@H](C)[C@H](C)CC5)([H])C(C)1C |
|  | 2α-hydroxybetulinic acid | C_30_H_48_O_4_ | 472.70 | 12305768 | O[C@H]1[C@H](O)C(C)(C)[C@@](CC[C@]2(C)[C@]3([H])CC[C@@]4([H])[C@@]2(C)CC[C@]5(C(O)=O)[C@]4([H])[C@H](C(C)=C)CC5)([H])[C@]3(C)C1 |
|  | rutin | C_27_H_30_O_16_ | 610.52 | 5280805 | OC1=C(O)C=C(C2=C(O[C@@H]3O[C@H](CO[C@@H]4O[C@@H](C)[C@H](O)[C@@H](O)[C@H]4O)[C@@H](O)[C@@H](O)[C@H]3O)C(C(C(O)=CC(O)=C5)=C5O2)=O)C=C1 |
|  | isoquercetin | C_21_H_20_O_12_ | 464.38 | 5280804 | OC1=CC(O)=C(C(C(O[C@H]2[C@H](O)[C@@H](O)[C@H](O)[C@@H](CO)O2)=C(C3=CC=C(O)C(O)=C3)O4)=O)C4=C1 |
|  | quercetin | C_15_H_10_O_7_ | 302.24 | 5280343 | OC1=CC(O)=C(C(C(O)=C(C2=CC=C(O)C(O)=C2)O3)=O)C3=C1 |
|  | astragalin | C_21_H_20_O_11_ | 448.38 | 5282102 | OC1=CC(O)=C(C(C(O[C@@H]2O[C@H](CO)[C@@H](O)[C@H](O)[C@H]2O)=C(C3=CC=C(O)C=C3)O4)=O)C4=C1 |
|  | wogonin-7-β-D-glcuronide | C_22_H_20_O_11_ | 460.39 | 3084961 | OC1=C(C(C=C(C2=CC=CC=C2)O3)=O)C3=C(OC)C(O[C@@H]4O[C@H](C(O)=O)[C@@H](O)C(O)[C@H]4O)=C1 |
|  | egenine | C_20_H_19_NO_6_ | 369.37 | 605434 | CN([C@]1([H])[C@@]2([H])C(C=CC3=C4OCO3)=C4[C@@H](O)O2)CCC(C1=C5)=CC6=C5OCO6 |
|  | (-)bicuculline | C_20_H_17_NO_6_ | 367.35 | 185838 | O=C1C2=C3C(OCO3)=CC=C2[C@]([H])([C@]4([H])C(C=C(OCO5)C5=C6)=C6CCN4C)O1 |
| Lonicerae Japonicae Flos | 4-O-Caffeoylquinic acid | C_16_H_18_O_9_ | 354.31 | 5315599 | O=C(OC1[C@H](O)CC(O)(C(O)=O)C[C@H]1O)/C=C/C2=CC(O)=C(O)C=C2 |
|  | luteolin | C_15_H_10_O_6_ | 286.23 | 5280445 | OC1=CC(O)=C(C(C=C(C2=CC(O)=C(O)C=C2)O3)=O)C3=C1 |
|  | lonicerin | C_27_H_30_O_15_ | 594.52 | 5282152 | O=C1C=C(C2=CC=C(O)C(O)=C2)OC3=C1C(O)=CC(O[C@@H]4O[C@H](CO)[C@@H](O)[C@H](O)[C@H]4O[C@@H]5O[C@@H](C)[C@H](O)[C@@H](O)[C@H]5O)=C3 |
|  | 5-hydroxy-7,4'-dimethoxyflavone | C_17_H_14_O_5_ | 298.29 | 5281601 | O=C(C=C(C1=CC=C(OC)C=C1)OC2=C3)C2=C(O)C=C3OC |
|  | luteolin-7-O-a-D-glucoside | C_21_H_20_O_11_ | 448.38 | 5280637 | O=C(C=C(C1=CC(O)=C(O)C=C1)OC2=C3)C2=C(O)C=C3O[C@H](O[C@H](CO)[C@H]4O)[C@H](O)[C@H]4O |
|  | luteolin-7-O-β-D-glucoside | C_21_H_20_O_11_ | 448.38 | 5280637 | O=C(C=C(C1=CC(O)=C(O)C=C1)OC2=C3)C2=C(O)C=C3O[C@@H](O[C@H](CO)[C@@H]4O)[C@H](O)[C@H]4O |
|  | quercetin-3-o-β-D-glucoside | C_21_H_19_O_12_ | 463.37 | 25203368 | O=C1C(O[C@](O[C@@](C([H])([H])O[H])([H])[C@@]([H])2O[H])([H])[C@@](O[H])([H])[C@]2([H])O[H])=C(c3c([H])c([H])c(O[H])c(O[H])c3[H])Oc4c([H])c(O[H])c([H])c(O[H])c14 |
|  | hyperoside | C_21_H_20_O_12_ | 464.38 | 5281643 | O=C(C(C(O)=CC(O)=C1)=C1O2)C(O[C@H]3[C@H](O)[C@@H](O)[C@@H](O)[C@@H](CO)O3)=C2C4=CC=C(O)C(O)=C4 |
|  | corymbosin | C_19_H_18_O_7_ | 358.34 | 10970376 | O=C(C(C(O)=CC(OC)=C1)=C1O2)C=C2C3=CC(OC)=C(OC)C(OC)=C3 |
|  | chlorogenic | C_16_H_18_O_9_ | 354.31 | 1794427 | OC1=C(O)C=CC(/C=C/C(O[C@@H]2C[C@@](C(O)=O)(O)C[C@@H](O)[C@H]2O)=O)=C1 |
|  | isichlorogentic acid | C_16_H_18_O_9_ | 354.31 | 73081 | OC1=C(O)C=CC(/C=C/C(O[C@H]2[C@@H](OC(/C=C/C3=CC(O)=C(O)C=C3)=O)[C@H](O)C[C@](O)(C(O)=O)C2)=O)=C1 |
|  | 4,5-Dicaffeoylquinic acid | C_25_H_24_O_12_ | 516.45 | 5459216 | O=C(/C=C/C1=CC(O)=C(O)C=C1)O[C@@H]([C@H](O)C[C@@](C(O)=O)(O)C2)[C@@H]2OC(/C=C/C3=CC(O)=C(O)C=C3)=O |
|  | Isochlorogenic acid B | C_25_H_24_O_12_ | 516.45 | 5281780 | OC1=C(O)C=CC(/C=C/C(O[C@@H]2[C@H](OC(/C=C/C3=CC(O)=C(O)C=C3)=O)[C@H](O)C[C@](C)(C(O)=O)C2)=O)=C1 |
|  | cynarin | C_25_H_24_O_12_ | 516.45 | 5281769 | OC1=C(O)C=C(/C=C/C(OC2C(O)CC(OC(/C=C/C3=CC=C(O)C(O)=C3)=O)(C(O)=O)CC2O)=O)C=C1 |
|  | 5-Caffeoylquinic acid | C_16_H_18_O_9_ | 354.31 | 12310830 | O=C(O[C@H]1C[C@@](O)(C(O)=O)C[C@H](O)[C@@H]1O)/C=C/C2=CC(O)=C(O)C=C2 |
|  | caffeic acid | C_9_H_8_O_4_ | 180.16 | 689043 | OC1=CC(/C=C/C(O)=O)=CC=C1O |
|  | palmitic acid | C_16_H_32_O_2_ | 256.42 | 985 | CCCCCCCCCCCCCCCC(O)=O |
|  | nonacosane | C_29_H_60_ | 408.79 | 12409 | CCCCCCCCCCCCCCCCCCCCCCCCCCCCC |
|  | linoleny alcohol | C_18_H_32_O | 264.45 | 6436081 | OCCCCCCCC/C=C/C/C=C/C/C=C/CC |
|  | cedrol | C_15_H_26_O | 222.37 | 65575 | C[C@@H]1CC[C@]2([H])[C@@]1(CC[C@@]3(C)O)C[C@H]3C2(C)C |
|  | heneicosane | C_21_H_44_ | 296.57 | 12403 | CCCCCCCCCCCCCCCCCCCCC |
|  | beta linalyl alcohol | C_10_H_18_O | 154.25 | 6549 | C=CC(C)(O)CC/C=C(C)/C |

**S Table 2. Similar drugs of *Shuang-Huang-Lian* components in DrugBank**

| **Herb** | **Component** | **Accession Number of Similar drugs in DrugBank** | **Similarity coefficient** |
| --- | --- | --- | --- |
| Forsythiae Fructus | γ-terpinene | DB02914 | 0.608 |
| Scutellariae Radix | leucosceptoside A | [DB08322](http://www.drugbank.ca/drugs/DB08322) | 0.609 |
| Scutellariae Radix | forsythoside D | [DB02675](http://www.drugbank.ca/drugs/DB02675) | 0.631 |
| Scutellariae Radix | caryophyllene | [DB03050](http://www.drugbank.ca/drugs/DB03050) | 0.632 |
| Scutellariae Radix | caryophyllene | [DB07752](http://www.drugbank.ca/drugs/DB07752) | 0.632 |
| Scutellariae Radix | acteoside | [DB08322](http://www.drugbank.ca/drugs/DB08322) | 0.635 |
| Forsythiae Fructus | forsythoside A | [DB08322](http://www.drugbank.ca/drugs/DB08322) | 0.635 |
| Forsythiae Fructus | forsythosideB | [DB08322](http://www.drugbank.ca/drugs/DB08322) | 0.635 |
| Forsythiae Fructus | acteoside | [DB08322](http://www.drugbank.ca/drugs/DB08322) | 0.635 |
| Forsythiae Fructus | β-hydroxyacteoside | [DB03208](http://www.drugbank.ca/drugs/DB03208) | 0.636 |
| Forsythiae Fructus | cornoside | [DB01092](http://www.drugbank.ca/drugs/DB01092) | 0.639 |
| Forsythiae Fructus | 3-carene | [DB08127](http://www.drugbank.ca/drugs/DB08127) | 0.64 |
| Forsythiae Fructus | 4-carene | [DB01770](http://www.drugbank.ca/drugs/DB01770) | 0.64 |
| Forsythiae Fructus | cornoside | [DB01078](http://www.drugbank.ca/drugs/DB01078) | 0.641 |
| Forsythiae Fructus | Phillyrin | [DB00773](http://www.drugbank.ca/drugs/DB00773) | 0.644 |
| Forsythiae Fructus | pinoresinol-β-D-glucoside | [DB00773](http://www.drugbank.ca/drugs/DB00773) | 0.644 |
| Forsythiae Fructus | forsythialan B | [DB04200](http://www.drugbank.ca/drugs/DB04200) | 0.652 |
| Forsythiae Fructus | forsythialan B | [DB01094](http://www.drugbank.ca/drugs/DB01094) | 0.652 |
| Forsythiae Fructus | forsythialan A | [DB01094](http://www.drugbank.ca/drugs/DB01094) | 0.655 |
| Scutellariae Radix | beta-patchoulene | [DB01493](http://www.drugbank.ca/drugs/DB01493) | 0.656 |
| Forsythiae Fructus | forsythialan A | [DB04200](http://www.drugbank.ca/drugs/DB04200) | 0.656 |
| Scutellariae Radix | salidroside | [DB02675](http://www.drugbank.ca/drugs/DB02675) | 0.671 |
| Forsythiae Fructus | salidroside | [DB02675](http://www.drugbank.ca/drugs/DB02675) | 0.671 |
| Forsythiae Fructus | limonene | [DB08127](http://www.drugbank.ca/drugs/DB08127) | 0.673 |
| Forsythiae Fructus | limonene | [DB01770](http://www.drugbank.ca/drugs/DB01770) | 0.673 |
| Forsythiae Fructus | arctiin | [DB00773](http://www.drugbank.ca/drugs/DB00773) | 0.682 |
| Forsythiae Fructus | norlapachol | [DB01117](http://www.drugbank.ca/drugs/DB01117) | 0.685 |
| Forsythiae Fructus | matairesionoside | [DB00773](http://www.drugbank.ca/drugs/DB00773) | 0.685 |
| Forsythiae Fructus | oleanolic acid | [DB01493](http://www.drugbank.ca/drugs/DB01493) | 0.697 |
| Forsythiae Fructus | oleanolic acid | [DB01431](http://www.drugbank.ca/drugs/DB01431) | 0.701 |
| Forsythiae Fructus | ursolic acid | [DB01708](http://www.drugbank.ca/drugs/DB01708) | 0.706 |
| Forsythiae Fructus | ursolic acid | [DB02789](http://www.drugbank.ca/drugs/DB02789) | 0.706 |
| Forsythiae Fructus | geranial | [DB06912](http://www.drugbank.ca/drugs/DB06912) | 0.707 |
| Forsythiae Fructus | pinocarveol | [DB02273](http://www.drugbank.ca/drugs/DB02273) | 0.707 |
| Forsythiae Fructus | terpinen-4-ol | [DB01524](http://www.drugbank.ca/drugs/DB01524) | 0.71 |
| Forsythiae Fructus | terpinen-4-ol | [DB04704](http://www.drugbank.ca/drugs/DB04704) | 0.71 |
| Forsythiae Fructus | Phillygenol | [DB07087](http://www.drugbank.ca/drugs/DB07087) | 0.722 |
| Forsythiae Fructus | α-pinene | [DB08127](http://www.drugbank.ca/drugs/DB08127) | 0.723 |
| Forsythiae Fructus | α-pinene | [DB01770](http://www.drugbank.ca/drugs/DB01770) | 0.723 |
| Forsythiae Fructus | pinoresinol | [DB07087](http://www.drugbank.ca/drugs/DB07087) | 0.728 |
| Forsythiae Fructus | epipinoresinol | [DB07087](http://www.drugbank.ca/drugs/DB07087) | 0.728 |
| Forsythiae Fructus | mentha（p-Mentha-1,8-dien-6-ol） | [DB01526](http://www.drugbank.ca/drugs/DB01526) | 0.73 |
| Lonicerae Japonicae Flos | linoleny alcohol | [DB00132](http://www.drugbank.ca/drugs/DB00132) | 0.732 |
| Lonicerae Japonicae Flos | linoleny alcohol | [DB00154](http://www.drugbank.ca/drugs/DB00154) | 0.732 |
| Forsythiae Fructus | α-terpineol | [DB03696](http://www.drugbank.ca/drugs/DB03696) | 0.742 |
| Forsythiae Fructus | 2α-hydroxybetulinic acid | [DB01586](http://www.drugbank.ca/drugs/DB01586) | 0.742 |
| Forsythiae Fructus | 2α-hydroxybetulinic acid | [DB07690](http://www.drugbank.ca/drugs/DB07690) | 0.742 |
| Forsythiae Fructus | 2α-hydroxybetulinic acid | [DB03619](http://www.drugbank.ca/drugs/DB03619) | 0.742 |
| Forsythiae Fructus | 2α-hydroxybetulinic acid | [DB02659](http://www.drugbank.ca/drugs/DB02659) | 0.742 |
| Scutellariae Radix | rivularin | [DB01852](http://www.drugbank.ca/drugs/DB01852) | 0.746 |
| Forsythiae Fructus | wogonin-7-β-D- glcuronide | [DB01698](http://www.drugbank.ca/drugs/DB01698) | 0.749 |
| Scutellariae Radix | daucosterol | [DB06750](http://www.drugbank.ca/drugs/DB06750) | 0.756 |
| Scutellariae Radix | wogonin | [DB08230](http://www.drugbank.ca/drugs/DB08230) | 0.759 |
| Scutellariae Radix | skullcapflavoneⅡ | [DB01852](http://www.drugbank.ca/drugs/DB01852) | 0.759 |
| Forsythiae Fructus | myrtanol | [DB00825](http://www.drugbank.ca/drugs/DB00825) | 0.759 |
| Scutellariae Radix | baicalin | [DB01698](http://www.drugbank.ca/drugs/DB01698) | 0.76 |
| Forsythiae Fructus | myrtanol | [DB01637](http://www.drugbank.ca/drugs/DB01637) | 0.76 |
| Forsythiae Fructus | myrtanol | [DB07171](http://www.drugbank.ca/drugs/DB07171) | 0.76 |
| Scutellariae Radix | skullcapflavoneⅠ | [DB01852](http://www.drugbank.ca/drugs/DB01852) | 0.763 |
| Forsythiae Fructus | betulinic acid | [DB03619](http://www.drugbank.ca/drugs/DB03619) | 0.767 |
| Forsythiae Fructus | betulinic acid | [DB01586](http://www.drugbank.ca/drugs/DB01586) | 0.767 |
| Forsythiae Fructus | betulinic acid | [DB06777](http://www.drugbank.ca/drugs/DB06777) | 0.767 |
| Forsythiae Fructus | betulinic acid | [DB07690](http://www.drugbank.ca/drugs/DB07690) | 0.767 |
| Forsythiae Fructus | betulinic acid | [DB02659](http://www.drugbank.ca/drugs/DB02659) | 0.767 |
| Forsythiae Fructus | β-phellandrene | [DB02914](http://www.drugbank.ca/drugs/DB02914) | 0.771 |
| Forsythiae Fructus | safrole | [DB01509](http://www.drugbank.ca/drugs/DB01509) | 0.778 |
| Lonicerae Japonicae Flos | cedrol | [DB03926](http://www.drugbank.ca/drugs/DB03926) | 0.789 |
| Lonicerae Japonicae Flos | cedrol | [DB03882](http://www.drugbank.ca/drugs/DB03926) | 0.789 |
| Lonicerae Japonicae Flos | cedrol | [DB01530](http://www.drugbank.ca/drugs/DB01530) | 0.789 |
| Forsythiae Fructus | p-cymene | [DB01722](http://www.drugbank.ca/drugs/DB01722) | 0.793 |
| Forsythiae Fructus | cymene | [DB01722](http://www.drugbank.ca/drugs/DB01722) | 0.793 |
| Forsythiae Fructus | rengyol | [DB00825](http://www.drugbank.ca/drugs/DB00825) | 0.806 |
| Lonicerae Japonicae Flos | 4-O-caffeoylquinic acid | [DB08322](http://www.drugbank.ca/drugs/DB08322) | 0.806 |
| Forsythiae Fructus | piperitol | [DB01526](http://www.drugbank.ca/drugs/DB01526) | 0.809 |
| Lonicerae Japonicae Flos | isochlorogenic acid B | [DB08322](http://www.drugbank.ca/drugs/DB08322) | 0.809 |
| Lonicerae Japonicae Flos | chlorogenic | [DB08322](http://www.drugbank.ca/drugs/DB08322) | 0.816 |
| Lonicerae Japonicae Flos | 5-caffeoylquinic acid | [DB08322](http://www.drugbank.ca/drugs/DB08322) | 0.816 |
| Forsythiae Fructus | myrcenol | [DB02273](http://www.drugbank.ca/drugs/DB02273) | 0.824 |
| Lonicerae Japonicae Flos | isichlorogentic acid | [DB08322](http://www.drugbank.ca/drugs/DB08322) | 0.83 |
| Lonicerae Japonicae Flos | 4,5-dicaffeoylquinic acid | [DB08322](http://www.drugbank.ca/drugs/DB08322) | 0.83 |
| Lonicerae Japonicae Flos | luteolin-7-O-a-D-glucoside | [DB01698](http://www.drugbank.ca/drugs/DB01698) | 0.837 |
| Lonicerae Japonicae Flos | luteolin-7-O-β-D-glucosie | [DB01698](http://www.drugbank.ca/drugs/DB01698) | 0.837 |
| Lonicerae Japonicae Flos | lonicerin | [DB01698](http://www.drugbank.ca/drugs/DB01698) | 0.838 |
| Lonicerae Japonicae Flos | cynarin | [DB08322](http://www.drugbank.ca/drugs/DB08322) | 0.843 |
| Scutellariae Radix | 7-methoxybaicalein | [DB07352](http://www.drugbank.ca/drugs/DB07352) | 0.844 |
| Forsythiae Fructus | myrcene | [DB03050](http://www.drugbank.ca/drugs/DB03050) | 0.846 |
| Forsythiae Fructus | myrcene | [DB07752](http://www.drugbank.ca/drugs/DB07752) | 0.846 |
| Forsythiae Fructus | myrcene | [DB03050](http://www.drugbank.ca/drugs/DB03050) | 0.846 |
| Forsythiae Fructus | myrcene | [DB07752](http://www.drugbank.ca/drugs/DB07752) | 0.846 |
| Scutellariae Radix | baicalein | [DB07352](http://www.drugbank.ca/drugs/DB07352) | 0.862 |
| Scutellariae Radix | oroxylin | [DB07352](http://www.drugbank.ca/drugs/DB07352) | 0.862 |
| Scutellariae Radix | butylated hydroxyanisole | [DB07726](http://www.drugbank.ca/drugs/DB07726) | 0.867 |
| Scutellariae Radix | norwogonin | [DB07352](http://www.drugbank.ca/drugs/DB07352) | 0.875 |
| Scutellariae Radix | menthone | [DB03540](http://www.drugbank.ca/drugs/DB03540) | 0.893 |
| Scutellariae Radix | menthone | [DB01744](http://www.drugbank.ca/drugs/DB01744) | 0.893 |
| Scutellariae Radix | isomenthone | [DB03540](http://www.drugbank.ca/drugs/DB03540) | 0.893 |
| Scutellariae Radix | isomenthone | [DB01744](http://www.drugbank.ca/drugs/DB01744) | 0.893 |
| Scutellariae Radix | isoborneol | [DB00825](http://www.drugbank.ca/drugs/DB00825) | 0.897 |
| Lonicerae Japonicae Flos | corymbosin | [DB08230](http://www.drugbank.ca/drugs/DB08230) | 0.897 |
| Forsythiae Fructus | β-amyrin acetate | [DB01493](http://www.drugbank.ca/drugs/DB01493) | 0.914 |
| Scutellariae Radix | 5,7,2'-trihydroxyflavone | [DB07352](http://www.drugbank.ca/drugs/DB07352) | 0.915 |
| Scutellariae Radix | dihydrooroxylinA | [DB01094](http://www.drugbank.ca/drugs/DB01094) | 0.924 |
| Lonicerae Japonicae Flos | 5-hydroxy-7,4'-dimethoxyflavone | [DB07352](http://www.drugbank.ca/drugs/DB07352) | 0.924 |
| Scutellariae Radix | 3,5,7,2'-tetrahydroxyflavone | [DB01852](http://www.drugbank.ca/drugs/DB01852) | 0.951 |
| Scutellariae Radix | neobaicalein | [DB08517](http://www.drugbank.ca/drugs/DB08517) | 0.958 |
| Forsythiae Fructus | astragalin | [DB01698](http://www.drugbank.ca/drugs/DB01698) | 0.966 |
| Scutellariae Radix | dihydrobaicalein | [DB01094](http://www.drugbank.ca/drugs/DB01094) | 0.967 |
| Scutellariae Radix | chrysin | [DB07352](http://www.drugbank.ca/drugs/DB07352) | 0.983 |
| Forsythiae Fructus | arctigenin | [DB04200](http://www.drugbank.ca/drugs/DB04200) | 0.991 |
| Forsythiae Fructus | dimethylmatairesinol | [DB04200](http://www.drugbank.ca/drugs/DB04200) | 0.991 |
| Forsythiae Fructus | isoquercetin | [DB01698](http://www.drugbank.ca/drugs/DB01698) | 0.991 |
| Lonicerae Japonicae Flos | quercetin-3-o-β-D-glucoside | [DB01698](http://www.drugbank.ca/drugs/DB01698) | 0.991 |
| Lonicerae Japonicae Flos | hyperoside | [DB01698](http://www.drugbank.ca/drugs/DB01698) | 0.991 |
| Scutellariae Radix | eriodictyol | [DB01094](http://www.drugbank.ca/drugs/DB01094) | 1 |
| Scutellariae Radix | acetophenone | [DB04619](http://www.drugbank.ca/drugs/DB04619) | 1 |
| Scutellariae Radix | guanine | [DB02377](http://www.drugbank.ca/drugs/DB02377) | 1 |
| Scutellariae Radix | phenethyl alcohol | [DB02192](http://www.drugbank.ca/drugs/DB02192) | 1 |
| Scutellariae Radix | octadecanoic acid | [DB03193](http://www.drugbank.ca/drugs/DB03193) | 1 |
| Scutellariae Radix | β-Sitosterol | [DB04540](http://www.drugbank.ca/drugs/DB04540) | 1 |
| Scutellariae Radix | β-Sitosterol | [DB04705](http://www.drugbank.ca/drugs/DB04705) | 1 |
| Scutellariae Radix | stigmasterol | [DB04540](http://www.drugbank.ca/drugs/DB04540) | 1 |
| Scutellariae Radix | stigmasterol | [DB04705](http://www.drugbank.ca/drugs/DB04705) | 1 |
| Forsythiae Fructus | camphor | [DB01744](http://www.drugbank.ca/drugs/DB01744) | 1 |
| Forsythiae Fructus | camphor | [DB03540](http://www.drugbank.ca/drugs/DB03540) | 1 |
| Forsythiae Fructus | 2-pentadecanone | [DB08688](http://www.drugbank.ca/drugs/DB08688) | 1 |
| Forsythiae Fructus | borneol | [DB04501](http://www.drugbank.ca/drugs/DB04501) | 1 |
| Forsythiae Fructus | matairesinol | [DB04200](http://www.drugbank.ca/drugs/DB04200) | 1 |
| Forsythiae Fructus | rutin | [DB01698](http://www.drugbank.ca/drugs/DB01698) | 1 |
| Forsythiae Fructus | quercetin | [DB02375](http://www.drugbank.ca/drugs/DB02375) | 1 |
| Forsythiae Fructus | quercetin | [DB04216](http://www.drugbank.ca/drugs/DB04216) | 1 |
| Lonicerae Japonicae Flos | luteolin | [DB08230](http://www.drugbank.ca/drugs/DB08230) | 1 |
| Lonicerae Japonicae Flos | caffeic acid | [DB01880](http://www.drugbank.ca/drugs/DB01880) | 1 |
| Lonicerae Japonicae Flos | palmitic acid | [DB03796](http://www.drugbank.ca/drugs/DB03796) | 1 |
| Lonicerae Japonicae Flos | palmitic acid | [DB03796](http://www.drugbank.ca/drugs/DB03796) | 1 |
| Lonicerae Japonicae Flos | nonacosane | [DB02440](http://www.drugbank.ca/drugs/DB02440) | 1 |
| Lonicerae Japonicae Flos | nonacosane | [DB03715](http://www.drugbank.ca/drugs/DB03715) | 1 |
| Lonicerae Japonicae Flos | nonacosane | [DB03563](http://www.drugbank.ca/drugs/DB03563) | 1 |
| Lonicerae Japonicae Flos | nonacosane | [DB02771](http://www.drugbank.ca/drugs/DB02771) | 1 |
| Lonicerae Japonicae Flos | nonacosane | [DB02826](http://www.drugbank.ca/drugs/DB02826) | 1 |
| Lonicerae Japonicae Flos | n-heneicosane | [DB02440](http://www.drugbank.ca/drugs/DB02440) | 1 |
| Lonicerae Japonicae Flos | n-heneicosane | [DB03715](http://www.drugbank.ca/drugs/DB03715) | 1 |
| Lonicerae Japonicae Flos | n-heneicosane | [DB03563](http://www.drugbank.ca/drugs/DB03563) | 1 |
| Lonicerae Japonicae Flos | n-heneicosane | [DB02771](http://www.drugbank.ca/drugs/DB02771) | 1 |
| Lonicerae Japonicae Flos | n-heneicosane | [DB02826](http://www.drugbank.ca/drugs/DB02826) | 1 |

**S Table 3. Components in PPI network**

| **Herb** | **Component** | **Molecular Formula** | **Molecular Weight** | **PubChem ID** | **Smiles string** |
| --- | --- | --- | --- | --- | --- |
| Scutellariae Radix | baicalin | C_21_H_18_O_11_ | 446.35 | 64982 | OC([C@@H]1[C@@H](O)[C@H](O)[C@@H](O)[C@H](OC2=C(C(O)=C3C(OC(C4=CC=CC=C4)=CC3=O)=C2)O)O1)=O |
|  | baicalein | C_15_H_10_O_5_ | 270.24 | 5281605 | OC1=C(OC)C(O)=C(C(C=C(C3=CC=CC=C3)O2)=O)C2=C1 |
|  | wogonoside | C_22_H_20_O_11_ | 460.39 | 29927693 | OC([C@@H](O)[C@@H](O)[C@H](O)C(C(C)=O)(C1=C(OC)C(OC(C2=CC=CC=C2)=CC3=O)=C3C(O)=C1)O)=O |
|  | wogonin | C_16_H_12_O_5_ | 284.26 | 5281703 | COC1=C(OC)C(OC(C3=CC=CC=C3)=CC2=O)=C2C(OC)=C1 |
|  | neobaicalein | C_17_H_14_O_6_ | 314.29 | 5320399 | O=C(C2)C(C(O)=CC(OC)=C1)=C1OC2C(C(O)=CC=C3)=C3O |
|  | skullcapflavoneⅡ | C_19_H_18_O_8_ | 374.34 | 124211 | COC1=C(C(O)=C2C(C=C(OC2=C1OC)C3=C(C=CC=C3OC)O)=O)OC |
|  | oroxylin，oroxylinA | C_16_H_12_O_5_ | 284.26 | 5320315 | OC1=C(OC)C(O)=C(C(C=C(C3=CC=CC=C3)O2)=O)C2=C1 |
|  | dihydrooroxylinA | C_16_H_14_O_5_ | 286.3 | 177032 | OC1=C(OC)C(O)=C(C(CC(C3=CC=CC=C3)(C)O2)=O)C2=C1 |
|  | chrysin | C_15_H_10_O_4_ | 254.24 | 5281607 | O=C(C(C(O)=CC(O)=C1)=C1O2)C=C2C3=CC=CC=C3 |
|  | skullcapflavoneⅠ | C_17_H_14_O_6_ | 314.29 | 5320399 | OC1=C2C(OC(C3=C(O)C=CC=C3)=CC2=O)=C(OC)C(OC)=C1 |
|  | 7-methoxybaicalein | C_16_H_12_O_5_ | 284.27 | 6226 | O=C2C1=C(O)C(O)=C(OC)C=C1OC(C3=CC=CC=C3)=C2 |
|  | norwogonin | C_15_H_10_O_5_ | 270.24 | 5281674 | OC1=C2C(OC(C3=CC=CC=C3)=CC2=O)=C(C(O)=C1)O |
|  | dihydrobaicalein | C_15_H_12_O_5_ | 272.25 | 9816931 | OC1=C(C(O)=C2C(CC(OC2=C1)C3=CC=CC=C3)=O)O |
|  | eriodictyol | C_15_H_12_O_6_ | 288.25 | 440735 | O=C1C(C(O)=CC(O)=C2)=C2O[C@H](C3=CC(O)=C(O)C=C3)C1 |
|  | rivularin | C_18_H_16_O_7_ | 344.32 | 13889022 | COC1=CC=CC(O)=C1C(OC2=C3C(O)=CC(OC)=C2OC)=CC3=O |
|  | leucosceptosideA | C_36_H_48_O_19_ | 784.75 | 45027865 | OC1=C(OC)C=C(/C=C/C(O[C@H]2[C@H](OC3O[C@@H](C)[C@H](O)[C@@H](O)[C@H]3O)[C@@H](O)[C@H](OCCC4=CC=C(O)C(O)=C4)O[C@@H]2CO)=O)C=C1 |
|  | acteoside | C_29_H_36_O_15_ | 624.59 | 5281800 | OC1=CC=C(C=C1O)/C=C/C(O[C@@H]2[C@@H](CO)O[C@@H](OCCC3=CC(O)=C(C=C3)O)[C@H](O)C2OC4O[C@@H](C)[C@H](O)[C@@H](O)[C@H]4O)=O |
|  | isomartynoside | C_31_H_40_O_15_ | 652.64 | 6476337 | OC1=C(OC)C=CC(/C=C/C(OC[C@H]2O[C@@H](OCCC3=CC(OC)=C(O)C=C3)[C@H](O)[C@@H](O[C@@H]4O[C@@H](C)[C@H](O)[C@@H](O)[C@H]4O)[C@@H]2O)=O)=C1 |
|  | salidroside | C_14_H_20_O_7_ | 300.3 | 159278 | O[C@@H]1[C@@H](O)[C@H](O)[C@@H](CO)O[C@H]1OCCC2=CC=C(O)C=C2 |
|  | isoprene | C_5_H_8_ | 68.12 | 6557 | CC(C=C)=C |
|  | acetophenone | C_8_H_8_O | 120.15 | 7410 | CC(C1=CC=CC=C1)=O |
|  | menthone | C_10_H_18_O | 154.25 | 26447 | O=C1C[C@@H](CC[C@H]1C(C)C)C |
|  | isomenthone | C_10_H_18_O | 154.25 | 6986 | O=C1CC(C)CCC1C(C)C |
|  | beta-patchoulene | C_15_H_24_ | 204.35 | 101731 | CC(C(C1)CC2)(C)C2(C3=C1C(C)CC3)C |
|  | guanine | C_5_H_5_N_5_O | 151.13 | 764 | NC(N1)=NC(N=CN2)=C2C1=O |
|  | 2-tert-butyl-4-methoxyphenol | C_11_H_16_O_2_ | 180.24 | 8456 | OC1=CC=C(OC)C=C1C(C)(C)C |
|  | phenethyl alcohol | C_8_H_10_O | 122.16 | 6054 | OCCC1=CC=CC=C1 |
|  | isoborneol | C_10_H_18_O | 154.25 | 6321405 | CC1([C@@]2(C)CC[C@](C[C@@H]2O)1[H])C |
|  | caryophyllene | C_15_H_24_ | 204.35 | 5322111 | C/C(CCC1C(C)(C)CC12)=C\CCC2=C |
|  | octadecanoic acid | C_18_H_36_O_2_ | 284.48 | 5281 | CCCCCCCCCCCCCCCCCC(O)=O |
|  | quebrachol | C_29_H_50_O | 414.71 | 222284 | CC(C)[C@@](CC)([H])CC[C@@]([H])(C)[C@@]4([H])CC[C@@]3([H])[C@]2([H])CC=C1C[C@](O)([H])CC[C@@](C)1[C@]([H])2CC[C@@]34C |
|  | stigmasterol | C_29_H_48_O | 412.69 | 412.69 | C[C@@]2(C(C(C)/C=C/[C@H](CC)C(C)C)CC3)C3C1CC=C(C4)[C@@](CC[C@@H]4O)(C)C1CC2 |
|  | daucosterol | C_35_H_60_O_6_ | 576.85 | 5742590 | O[C@@H]1[C@@H](O)[C@H](O[C@@H]3CC2=CC[C@]([C@@]5([H])[C@]([C@]([C@H](C)CC[C@@H](CC)C(C)C)([H])CC5)(C)CC4)([H])[C@@]4([H])[C@](C)2CC3)O[C@H](CO)[C@H]1O.[O] |
|  | 3,5,7,2'-tetrahydroxyflavone | C_15_H_10_O_6_ | 286.24 | 5281610 | OC1=CC=CC(O)=C1C3=C(O)C(C2=C(O)C=C(O)C=C2O3)=O |
|  | 5,7,2'-trihydroxyflavone | C_15_H_10_O_5_ | 270.24 | 5322064 | O=C1C=C(C3=CC=CC=C3O)OC2=C1C(O)=CC(O)=C2 |
|  | β-Sitosterol | C_35_H_60_O_2_ | 512.85 | 489644 | CC(C)[C@@](CC)([H])CC[C@@]([H])(C)[C@@]4([H])CC[C@@]3([H])[C@]2([H])CC=C1C[C@](O)([H])CC[C@@](C)1[C@]([H])2CC[C@@]34C |
| Forsythiae Fructus | α-pinene | C_10_H_16_ | 136.23 | 6654 | CC1(C)C2CC=C(C)C1C2 |
|  | camphene | C_10_H_16_ | 136.23 | 6616 | CC1(C)C2CCC(C2)C1=C |
|  | β-pinene | C_10_H_16_ | 136.23 | 14896 | CC1(C)C(CC2)([H])CC([H])1C2=C |
|  | p-cymene | C_10_H_14_ | 134.22 | 7463 | CC1=CC=C(C(C)C)C=C1 |
|  | limonene | C_10_H_16_ | 136.23 | 22311 | CC1=CC[C@H]([C@@](C)=C)CC1 |
|  | γ-terpinene | C_10_H_16_ | 136.23 | 7461 | CC1=CCC(C(C)C)=CC1 |
|  | β-phellandrene | C_10_H_16_ | 136.23 | 11142 | CC(C)C(CC1)C=CC1=C |
|  | myrcene | C_10_H_16_ | 136.23 | 31253 | C/C(C)=C/CCC(C=C)=C |
|  | β-ocimene | C_10_H_16_ | 136.23 | 5281553 | C/C(C)=C/C/C=C(C)/C=C |
|  | 3-carene | C_10_H_16_ | 136.23 | 26049 | CC1=CCC2C(C(C)2C)C1 |
|  | cymene | C_10_H_14_ | 134.22 | 10812 | CC1=CC=C(C(C)C)C=C1 |
|  | carveol（p-Mentha-1,8-dien-6-ol） | C_10_H_16_O | 152.23 | 7438 | CC(C1CC=C(C)C(O)C1)=C |
|  | myrcene | C_10_H_16_ | 136.23 | 31253 | C/C(C)=C/CCC(C=C)=C |
|  | camphor | C_10_H_16_O | 152.23 | 2537 | CC1(C2=O)C(C)(C)C(C2)CC1 |
|  | geranial | C_10_H_16_O | 152.23 | 638011 | C/C(C)=C/CC/C(C)=C/C=O |
|  | 2-pentadecanone | C_15_H_30_O | 226.4 | 61303 | CCCCCCCCCCCCCC(C)=O |
|  | borneol | C_10_H_18_O | 154.25 | 64685 | C[C@@]12[C@@H](C)C[C@@H]([C@@](C)2C)CC1 |
|  | α-terpineol | C_10_H_18_O | 154.25 | 17100 | CC1=CCC(C(C)(O)C)CC1 |
|  | safrole | C_10_H_10_O_2_ | 162.19 | 5144 | C=CCC1=CC=C2C(OCO2)=C1 |
|  | linalool | C_10_H_18_O | 154.25 | 6549 | C/C(C)=C\CCC(C=C)(O)C |
|  | terpinen-4-ol | C_10_H_18_O | 154.25 | 11230 | CC1=CCC(O)(C(C)C)CC1 |
|  | norlapachol | C_14_H_12_O_3_ | 228.24 | 231114 | OC2=C(\C=C(C)/C)C(C1=C(C2=O)C=CC=C1)=O |
|  | pinocarveol | C_10_H_16_O | 152.23 | 102667 | OC1C(C(C2)C(C)(C)C2C1)=C |
|  | myrcenol | C_10_H_18_O | 154.25 | 10975 | C=CC(CCCC(C)(O)C)=C |
|  | myrtanol | C_10_H_18_O | 154.25 | 521314 | CC12CCC(C)(CO)C(C)(C2)C(C)1C |
|  | piperitol | C_10_H_18_O | 154.25 | 10282 | CC(C)C1CCC(C)=CC1O |
|  | methyl tetracosanoate | C_25_H_50_O_2_ | 382.66 | 75546 | CCCCCCCCCCCCCCCCCCCCCCCC(=O)OC |
|  | cornoside | C_14_H_20_O_8_ | 3084796 | 3084796 | C1=CC(C=CC1=O)(CCO[C@@H]2[C@H]([C@@H]([C@H]([C@@H](O2)CO)O)O)O)O |
|  | rengyol | C_8_H_16_O_3_ | 160.21 | 363707 | OC1(CCO)CCC(O)CC1 |
|  | salidroside | C_14_H_20_O_7_ | 300.3 | 159278 | O[C@@H]1[C@@H](CO)O[C@@H](OCCC2=CC=C(O)C=C2)[C@H](O)[C@@H]1O |
|  | forsythosideA(suspensaside) | C_29_H_36_O_15_ | 624.59 | 45358127 | OC1=CC=C(/C=C/C(O[C@H]2[C@H](O)[C@@H](O)[C@H](OCCC4=CC(O)=C(O)C=C4)O[C@@H]2CO[C@H]3[C@H](O)[C@H](O)[C@@H](O)[C@H](C)O3)=O)C=C1O |
|  | forsythosideB | C_34_H_44_O_19_ | 756.7 | 23928102 | OC1=C(O)C=C(CCO[C@H]2[C@H](O)[C@@H](O)[C@H](OC(/C=C/C4=CC(O)=C(O)C=C4)=O)[C@@H](CO[C@@H]3O[C@@H](C)[C@H](O)[C@@H](O)[C@H]3O)O2)C=C1 |
|  | β-Hydroxyacteoside | C_29_H_36_O_16_ | 640.59 | 10009317 | OC1=CC(/C=C/C(O[C@H]2[C@H](O[C@@H]3O[C@@H](C)[C@H](O)[C@@H](O)[C@H]3O)[C@@H](O)[C@H](OCC(O)C4=CC=C(O)C(O)=C4)O[C@@H]2CO)=O)=CC=C1O |
|  | acteoside | C_29_H_36_O_15_ | 624.59 | 5281800 | OC1=C(O)C=C(/C=C/C(O[C@H]2[C@H](OC3O[C@@H](C)[C@H](O)[C@@H](O)[C@H]3O)[C@@H](O)[C@H](OCCC4=CC(O)=C(O)C=C4)O[C@@H]2CO)=O)C=C1 |
|  | arctigenin | C_21_H_24_O_6_ | 372.41 | 64981 | COC1=CC=C(C[C@@H]2[C@@H](CC3=CC=C(O)C(OC)=C3)C(OC2)=O)C=C1OC |
|  | arctiin | C_27_H_34_O_11_ | 534.55 | 100528 | CC[C@@H]1[C@@H](O)[C@H](O)[C@@H](O)[C@H](OC2=C(OC)C=C(C[C@H]3C(OC[C@@H]3CC4=CC(OC)=C(OC)C=C4)=O)C=C2)O1 |
|  | matairesinol | C_20_H_22_O_6_ | 358.39 | 119205 | COC1=CC(C[C@@H]2[C@@H](CC3=CC=C(O)C(OC)=C3)COC2=O)=CC=C1O |
|  | matairesionoside | C_26_H_32_O_11_ | 520.53 | 486612 | COC1=C(O[C@@H]3O[C@H](CO)[C@@H](O)[C@H](O)[C@H]3O)C=CC([C@H]([H])C2[C@@]([H])(CC4=CC=C(O)C(OC)=C4)COC2=O)=C1 |
|  | dimethylmatairesinol | C_22_H_26_O_6_ | 386.44 | 1286 | COC1=C(OC)C=CC(CC2C(CC3=CC=C(OC)C(OC)=C3)COC2=O)=C1 |
|  | phillyrin | C_27_H_34_O_11_ | 534.55 | 44584288 | O[C@@H]1[C@@H](O)[C@H](OC2=CC=[C@@]([C@H]3OC[C@@]4([H])[C@@]([H])3CO[C@H]4[C@]5=CC(OC)=C(OC)C=C5)C=C2OC)O[C@H](CO)[C@H]1O |
|  | phillygenol | C_21_H_24_O_6_ | 372.41 | 3083590 | COC1=C(OC)C=[C@@]([C@@H]2OC[C@@]3([H])[C@@]([H])2CO[C@@H]3[C@]4=CC(OC)=C(O)C=C4)C=C1 |
|  | pinoresinol | C_20_H_22_O_6_ | 358.39 | 234817 | OC1=C(OC)C=[C@@]([C@@H]2OC[C@@]3([H])[C@@]([H])2CO[C@H]3[C@]4=CC(OC)=C(O)C=C4)C=C1 |
|  | pinoresinol-β-D-glucoside | C_26_H_32_O_11_ | 520.53 | 486614 | O[C@@H]1[C@@H](O)[C@H](OC2=CC=[C@@]([C@H]3OC[C@@]4([H])[C@@]([H])3CO[C@H]4[C@]5=CC(OC)=C(O)C=C5)C=C2OC)O[C@H](CO)[C@H]1O |
|  | epipinoresinol | C_20_H_22_O_6_ | 358.39 | 637584 | OC1=CC=[C@]([C@@H]2[C@@H](CO[C@H]3[C@]4=CC=C(O)C(OC)=C4)[C@@H]3CO2)C=C1OC |
|  | forsythialan A | C_20_H_22_O_7_ | 374.38 | 44453412 | OC1=CC=C(C([C@]2([H])[C@]([H])(CO)[C@@H]([C@]3=CC(O)=CC(OC)=C3)OC2)=O)C=C1OC |
|  | forsythialan B | C_20_H_22_O_7_ | 388.41 | 44453332 | COC1=CC(O)=C[C@]([C@H]2OC[C@]([H])(C(C3=CC=C(OC)C(OC)=C3)=O)[C@](CO)2[H])=C1 |
|  | betulinic acid | C_30_H_48_O_3_ | 456.7 | 64971 | O[C@H]1CC[C@@]2(C)[C@](CC[C@]3(C)[C@@]([H])2CC[C@]4([H])[C@](C)3CC[C@]5([C@](O)=O)[C@@]([H])4[C@H]([C@@](C)=C)CC5)([H])C(C)1C |
|  | oleanolic acid | C_30_H_48_O_3_ | 456.7 | 10494 | O[C@H]1CC[C@@]2(C)[C@](CC[C@]3(C)[C@@]([H])2CC=C4[C@](C)3CC[C@]5([C@](O)=O)[C@]([H])4CC(C)(C)CC5)([H])C(C)1C |
|  | β-amyrin acetate (beta-Amyrenol) | C_30_H_50_O | 426.72 | 73145 | O[C@H]1CC[C@@]2(C)[C@](CC[C@]3(C)[C@@]([H])2CC=C4[C@](C)3CC[C@]5(C)[C@]([H])4CC(C)(C)CC5)([H])C(C)1C |
|  | ursolic acid | C_30_H_48_O_3_ | 456.7 | 64945 | O[C@H]1CC[C@@]2(C)[C@](CC[C@]3(C)[C@@]([H])2CC=C4[C@](C)3CC[C@]5([C@@](O)=O)[C@]([H])4[C@@H](C)[C@H](C)CC5)([H])C(C)1C |
|  | 2α-hydroxybetulinic acid | C_30_H_48_O_4_ | 472.70 | 12305768 | O[C@H]1[C@H](O)C(C)(C)[C@@](CC[C@]2(C)[C@]3([H])CC[C@@]4([H])[C@@]2(C)CC[C@]5(C(O)=O)[C@]4([H])[C@H](C(C)=C)CC5)([H])[C@]3(C)C1 |
|  | rutin | C_27_H_30_O_16_ | 610.52 | 5280805 | OC1=C(O)C=C(C2=C(O[C@@H]3O[C@H](CO[C@@H]4O[C@@H](C)[C@H](O)[C@@H](O)[C@H]4O)[C@@H](O)[C@@H](O)[C@H]3O)C(C(C(O)=CC(O)=C5)=C5O2)=O)C=C1 |
|  | isoquercetin | C_21_H_20_O_12_ | 464.38 | 5280804 | OC1=CC(O)=C(C(C(O[C@H]2[C@H](O)[C@@H](O)[C@H](O)[C@@H](CO)O2)=C(C3=CC=C(O)C(O)=C3)O4)=O)C4=C1 |
|  | quercetin | C_15_H_10_O_7_ | 302.24 | 5280343 | OC1=CC(O)=C(C(C(O)=C(C2=CC=C(O)C(O)=C2)O3)=O)C3=C1 |
|  | astragalin | C_21_H_20_O_11_ | 448.38 | 5282102 | OC1=CC(O)=C(C(C(O[C@@H]2O[C@H](CO)[C@@H](O)[C@H](O)[C@H]2O)=C(C3=CC=C(O)C=C3)O4)=O)C4=C1 |
|  | wogonin-7-β-D-glcuronide | C_22_H_20_O_11_ | 460.39 | 3084961 | OC1=C(C(C=C(C2=CC=CC=C2)O3)=O)C3=C(OC)C(O[C@@H]4O[C@H](C(O)=O)[C@@H](O)C(O)[C@H]4O)=C1 |
|  | egenine | C_20_H_19_NO_6_ | 369.37 | 605434 | CN([C@]1([H])[C@@]2([H])C(C=CC3=C4OCO3)=C4[C@@H](O)O2)CCC(C1=C5)=CC6=C5OCO6 |
|  | (-)bicuculline | C_20_H_17_NO_6_ | 367.35 | 185838 | O=C1C2=C3C(OCO3)=CC=C2[C@]([H])([C@]4([H])C(C=C(OCO5)C5=C6)=C6CCN4C)O1 |
|  | (-)-7′-O-methylegenine | C_21_H_21_NO_6_ | 383.39 | 197775 | CN1[C@]([C@]2([H])C(C=CC3=C4OCO3)=C4C(OC)O2)([H])C5=CC6=C(OCO6)C=C5CC1 |
|  | suspensine A | C_19_H_17_NO_4_ | 323.34 | 6770 | CN1CC2=C(C=CC3=C2OCO3)CC4=CC5=C(OCO5)C=C4C1 |
|  | forsythoside D | C_19_H_28_O_11_ | 432.42 | 192437 | OC1=C(O)C=C(C(CO[C@H]2[C@H](O)[C@@H](O)[C@H](O)[C@@H](CO[C@H]3[C@H](O)[C@H](O)[C@@H](O)[C@H](C)O3)O2)([H])O)C=C1 |
| Lonicerae Japonicae Flos | luteolin | C_15_H_10_O_6_ | 286.23 | 5280445 | OC1=CC(O)=C(C(C=C(C2=CC(O)=C(O)C=C2)O3)=O)C3=C1 |
|  | lonicerin | C_27_H_30_O_15_ | 594.52 | 5282152 | O=C1C=C(C2=CC=C(O)C(O)=C2)OC3=C1C(O)=CC(O[C@@H]4O[C@H](CO)[C@@H](O)[C@H](O)[C@H]4O[C@@H]5O[C@@H](C)[C@H](O)[C@@H](O)[C@H]5O)=C3 |
|  | luteolin-7-O-a-D-glucoside | C_21_H_20_O_11_ | 448.38 | 5280637 | O=C(C=C(C1=CC(O)=C(O)C=C1)OC2=C3)C2=C(O)C=C3O[C@H](O[C@H](CO)[C@H]4O)[C@H](O)[C@H]4O |
|  | luteolin-7-O-β-D-glucoside | C_21_H_20_O_11_ | 448.38 | 5280637 | O=C(C=C(C1=CC(O)=C(O)C=C1)OC2=C3)C2=C(O)C=C3O[C@@H](O[C@H](CO)[C@@H]4O)[C@H](O)[C@H]4O |
|  | quercetin-3-o-β-D-glucoside | C_21_H_19_O_12_ | 463.37 | 25203368 | O=C1C(O[C@](O[C@@](C([H])([H])O[H])([H])[C@@]([H])2O[H])([H])[C@@](O[H])([H])[C@]2([H])O[H])=C(c3c([H])c([H])c(O[H])c(O[H])c3[H])Oc4c([H])c(O[H])c([H])c(O[H])c14 |
|  | hyperoside | C_21_H_20_O_12_ | 464.38 | 5281643 | O=C(C(C(O)=CC(O)=C1)=C1O2)C(O[C@H]3[C@H](O)[C@@H](O)[C@@H](O)[C@@H](CO)O3)=C2C4=CC=C(O)C(O)=C4 |
|  | corymbosin | C_19_H_18_O_7_ | 358.34 | 10970376 | O=C(C(C(O)=CC(OC)=C1)=C1O2)C=C2C3=CC(OC)=C(OC)C(OC)=C3 |
|  | chlorogenic | C_16_H_18_O_9_ | 354.31 | 1794427 | OC1=C(O)C=CC(/C=C/C(O[C@@H]2C[C@@](C(O)=O)(O)C[C@@H](O)[C@H]2O)=O)=C1 |
|  | isichlorogentic acid | C_16_H_18_O_9_ | 354.31 | 73081 | OC1=C(O)C=CC(/C=C/C(O[C@H]2[C@@H](OC(/C=C/C3=CC(O)=C(O)C=C3)=O)[C@H](O)C[C@](O)(C(O)=O)C2)=O)=C1 |
|  | 4,5-Dicaffeoylquinic acid | C_25_H_24_O_12_ | 516.45 | 5459216 | O=C(/C=C/C1=CC(O)=C(O)C=C1)O[C@@H]([C@H](O)C[C@@](C(O)=O)(O)C2)[C@@H]2OC(/C=C/C3=CC(O)=C(O)C=C3)=O |
|  | Isochlorogenic acid B | C_25_H_24_O_12_ | 516.45 | 5281780 | OC1=C(O)C=CC(/C=C/C(O[C@@H]2[C@H](OC(/C=C/C3=CC(O)=C(O)C=C3)=O)[C@H](O)C[C@](C)(C(O)=O)C2)=O)=C1 |
|  | cynarin | C_25_H_24_O_12_ | 516.45 | 5281769 | OC1=C(O)C=C(/C=C/C(OC2C(O)CC(OC(/C=C/C3=CC=C(O)C(O)=C3)=O)(C(O)=O)CC2O)=O)C=C1 |
|  | 5-Caffeoylquinic acid | C_16_H_18_O_9_ | 354.31 | 12310830 | O=C(O[C@H]1C[C@@](O)(C(O)=O)C[C@H](O)[C@@H]1O)/C=C/C2=CC(O)=C(O)C=C2 |
|  | caffeic acid | C_9_H_8_O_4_ | 180.16 | 689043 | OC1=CC(/C=C/C(O)=O)=CC=C1O |
|  | palmitic acid | C_16_H_32_O_2_ | 256.42 | 985 | CCCCCCCCCCCCCCCC(O)=O |
|  | nonacosane | C_29_H_60_ | 408.79 | 12409 | CCCCCCCCCCCCCCCCCCCCCCCCCCCCC |
|  | linoleny alcohol | C_18_H_32_O | 264.45 | 6436081 | OCCCCCCCC/C=C/C/C=C/C/C=C/CC |
|  | cedrol | C_15_H_26_O | 222.37 | 65575 | C[C@@H]1CC[C@]2([H])[C@@]1(CC[C@@]3(C)O)C[C@H]3C2(C)C |
|  | heneicosane | C_21_H_44_ | 296.57 | 12403 | CCCCCCCCCCCCCCCCCCCCC |
|  | beta linalyl alcohol | C_10_H_18_O | 154.25 | 6549 | C=CC(C)(O)CC/C=C(C)/C |
|  | 4-O-Caffeoylquinic acid | C_16_H_18_O_9_ | 354.31 | 5315599 | O=C(OC1[C@H](O)CC(O)(C(O)=O)C[C@H]1O)/C=C/C2=CC(O)=C(O)C=C2 |
|  | 5-hydroxy-7,4'-dimethoxyflavone | C_17_H_14_O_5_ | 298.29 | 5281601 | O=C(C=C(C1=CC=C(OC)C=C1)OC2=C3)C2=C(O)C=C3OC |
